# Modelling optimal lockdowns with waning immunity

**DOI:** 10.1007/s00199-022-01468-8

**Published:** 2022-11-29

**Authors:** Aditya Goenka, Lin Liu, Manh-Hung Nguyen

**Affiliations:** 1 Department of Economics, University of Birmingham, Birmingham, England; 2 Management School, University of Liverpool, Liverpool, England; 3 Toulouse School of Economics, INRAE, University of Toulouse Capitole, Toulouse, France

**Keywords:** Covid-19, *SIRS* model, Mortality, Lockdown, Quarantine, Sufficiency conditions, Self-isolation, Infectious diseases, NPI, Endogenous discounting, E13, E22, D15, D50, D63, I10, I15, I18, O41, C61

## Abstract

This paper studies continuing optimal lockdowns (can also be interpreted as quarantines or self-isolation) in the long run if a disease (Covid-19) is endemic and immunity can fail, that is, the disease has SIRS dynamics. We model how disease related mortality affects the optimal choices in a dynamic general equilibrium neoclassical growth framework. An extended welfare function that incorporates loss from mortality is used. In a disease endemic steady state, without this welfare loss even if there is continuing mortality, it is not optimal to impose even a partial lockdown. We characterize how the optimal restriction and equilibrium outcomes vary with the effectiveness of the lockdown, the productivity of working from home, the rate of mortality from the disease, and failure of immunity. We provide the sufficiency conditions for economic models with *SIRS* dynamics with disease related mortality–a class of models which are non-convex and have endogenous discounting so that no existing results are applicable.

We thank seminar participants in Birmingham, Durham, ISI Delhi, Kansas, Macau, NYUAD, and the T2M Conference 2022, the anonymous referee, and the Associate Editor for their helpful comments. The usual disclaimer applies. Manh-Hung Nguyen acknowledges support from ANR under grant ANR-17-EURE-0010 (Investissements d'Avenir program).

✉ Aditya Goenka
a.goenka@bham.ac.uk

Lin Liu
lin.liu@liverpool.ac.uk

Manh-Hung Nguyen
manh-hung.nguyen@tse-fr.eu

## 1 Introduction

The Covid-19 pandemic has brought the study of the interaction of epidemiology modelling and economic outcomes to the forefront of economic research. As there are no medical interventions to fully prevent and effectively treat Covid-19 there is an interest in the role of non-pharmaceutical interventions (NPI) to control the disease.[1] This paper studies optimal lockdowns, that is, where both the healthy (susceptible as well as recovered) and the infected (infectious) are quarantined. We model this in a neoclassical growth framework where the disease evolves according to $SIRS$ dynamics and there is mortality due to the disease. This is motivated by the fact that it is not well understood how long immunity lasts from a prior infection of Covid-19.[2] The SARS-Cov-2 virus is not a stable virus such as the ones that cause measles and small-pox, and which have been well-controlled by vaccination programs.[3] The new variants, especially the Variants of Concern-Beta, Delta and Omicron escape immunity conferred by earlier infections and existing vaccines are not fully effective against them.[4] For other viruses, immunity also declines or wanes (Cohen 2019). The interactions between variants and diseases is complex: a new variant can enhance immunity against earlier ones so that it becomes the dominant variant but may escape prior immunity conferred by earlier variants (Khan et al. 2022) and exposure to other diseases may facilitate the emergence of variants (Cele et al. 2021b). Thus, it is important for economists to model the emergence of the new strains or waning immunity as it will affect economic outcomes.[5]

In this paper, households can save by investing in capital, and production of the single consumption good uses capital and labor. Only the healthy (susceptible and the recovered) individuals can work. Motivated by Covid-19 we abstract from health expenditures that can be used for prevention and treatment[6] and the only way to

[1] The new anti-viral drugs developed to treat Covid are not widely used at the time of writing the paper. Corticosteroids do reduce mortality and these have been available for many years. See Siemieniuk (2021) for a review.

[2] Long et al. (2020) using data from China find evidence consistent with a steep decline in 2-3 months. Similar results were found in a study in the US (Ibarrando et al. 2020). On the other hand, Wajnberg et al. (2020) and Sekine et al. (2020) find evidence suggesting longer immunity.

[3] See https://nextstrain.org/ncov/global for mutation lineages of the SARS-Cov-2 virus.

[4] See Andrews et al. (2021), Cele et al. (2021a); Dejnirattisai et al. (2021); Garcia-Beltran (2021); Hansen et al. (2021); Sabine et al. (2021); Wibmer et al. (2021). Other variants such as Epsilon also escape immunity (Deng et al. 2021) but did not seed widely. The evidence on the Omicron variant shows that it escapes immunity from prior infections and vaccinations (Carreño et al. 2022; Cao et al. 2021; Cele et al. 2022; Rössler et al. 2022).

[5] Giannitsarou et al. (2021) also study the problem of mitigating a pandemic in a model with $SEIRS$ dynamics but the modelling strategy is different. They look at effect of social distancing in a partial equilibrium model where the flow utilities of being in different health states is constant. Their paper concentrates on short-run transitional dynamics whereas this paper uses a dynamic general equilibrium model analyzing steady states where the disease may be endemic.

[6] Goldman and Lightwood (2004) is an early paper studying expenditures for treatment in a partial equilibrium $SIS$ model. Goenka and Liu (2020), and Goenka et al. (2014, 2020) modelled optimal health expenditures in a growth framework similar to the current paper.

control the disease is by quarantines.[7] Goenka and Liu (2012) is an early contribution studying ad-hoc quarantines to reduce infections in a $SIS$ model. Quarantines are imperfect as a mechanism to control the disease as their effectiveness or compliance with them is not complete. The productivity of those quarantined is reduced, and the labor supply available for productive activity is the fraction of the healthy not quarantined plus the reduced productivity of the healthy quarantined. The productivity of all healthy who are quarantined drops but as there is only partial compliance, only a fraction of those quarantined do not circulate in the population and do not transmit infections. There is disease related mortality and a fraction of the infected die. The optimal quarantine decision can also be interpreted as the optimal decision to self-isolate. The distinction between self-isolation and a quarantine is that in the latter it is mandated rather than being an individual decision. The evidence suggests that even without mandated quarantines, self-isolation in response to infections takes place and in the paper we do not distinguish between the two interpretations.[8] In the model, the households are homogeneous and we do not model disease related externality where households do not take into account the effect of their decisions on the evolution of the disease in the population.[9]

The model is a fully dynamic general equilibrium model and we characterize the Euler equations that govern the evolution of the economy. As our interest is beyond the very short run, we show that there are two steady states for the economy: a disease free and disease endemic steady state. The extent of the optimal lockdown depends on a function of the parameters and the equilibrium values of the economic variables. The equilibrium reproduction rate, $R_0$, will depend on the characteristics of the disease (infectivity, recovery rate, mortality due to the disease, and failure of immunity), economic parameters as well as endogenous economic choices. The paper analyses whether quarantines will persist in the long run when the disease is endemic. In the paper we not analyse short-run dynamics, even though the model can allows for this analysis as the full dynamical system of the model is specified.[10]

In the pure utilitarian model where the welfare depends only on utility from consumption, even when the morbidity effects of the disease impacting labor supply is taken into account, it is not optimal to impose quarantines in a steady state. The mortality from the disease has small effects on the labor supply but the costs associated with lockdowns outweigh the gain from restricting mortality. However, it seems that many countries were explicitly motivated by public health concerns of disease related mortality in imposing of lockdowns during the Covid pandemic.[11] Some countries

[7] As there is homogeneous mixing in our model and we do not include the exposed, $E$, health state, a lockdown is equivalent to a lockdown. We will use them interchangeably.

[8] See Sheridan et al. (2020) and Zhang et al. (2021) for comparisons across Scandinavian countries as Sweden did not impose quarantines but Norway and Denmark did. Goolsbee and Svyerson (2020) present US evidence.

[9] This has been modelled in different ways in the literature. See for example, Gersovitz and Hammer (2004), Goenka and Liu (2020), and Toxvaerd (2019, 2020).

[10] The main reason is the curse of dimensionality as the dynamical system is four dimensional.

[11] For example, China, Denmark, New Zealand, Singapore, Vietnam, and Norway based their decisions primarily on public health advice.

seemed to be concerned about the trade-offs between health and wealth.[12] How will these trade-offs in the welfare function affect health and income outcomes in the longer run will be important in understanding the persistence of Covid and its longer run economic implications. Thus, we extend the utilitarian welfare function by including a loss in welfare from infections and mortality (the latter is a fixed fraction of infections). This has also been done in other papers.[13] We characterize the optimal quarantine using the extended welfare function and derive the steady states.

In the paper, the degree of compliance with lockdowns, the drop in productivity from working at home, the mortality rate from disease, the rate of escape from immunity, and the weight assigned to welfare loss from infections and mortality are treated as parameters and we study how the equilibrium economic and health outcomes vary with them. Varying these measures helps in understanding the differences in policy choices and resulting outcomes. First, as compliance with quarantines increases, the optimal quarantine first increases and then decreases reflecting the fact that the impact of quarantines is the product of magnitude of the quarantine and compliance with it. The increase in compliance eventually can be traded-off with a reduced quarantine. Second, with increased productivity from working from home, the cost of imposing a lockdown increases, and the extent of the optimal lockdown increases. Countries and social groups with lower ability to work from home will thus, be expected to have less severe lockdowns. This will manifest in higher infection and mortality rates reflecting the endogeniety of the policy response to the constraints on working from home. Third, the effect of increasing the mortality rate on the optimal quarantine is stricter quarantines. The stricter quarantine reduces reducing mortality at the cost of lower output, consumption, and capital stock. This response is largely driven by how the welfare loss from disease related mortality as in equilibrium, the extent of mortality is small and does not have significant effects on labor supply. Fourth, as the rate at which immunity fails increases, quarantines increase in a concave manner but infections first increase and then eventually decrease. If the contact rate is high, then in the long run even if infections and mortality persist, it is not optimal to impose quarantines as the fraction of recovered in the population is higher. Note that this is in a steady state and policies may differ along the transition path. Fifth, if there is a low weight assigned to the welfare loss from mortality then there are no quarantines. As the weight on welfare loss from mortality increases so does the severity of quarantine, leading to lower mortality and lower economic outcomes. There is an increase in the proportion of susceptible population but a decrease in the recovered. These results suggest that focussing on the degree of severity of a policy tracker as in the news and policy space can be misleading.

There is a growing literature on quarantines in economic epidemiology models. These papers generally look at short run transitional dynamics.[14] and generally do

[12] Most notably UK and USA.

[13] Acemoglu et al. (2021), Alvarez et al. (2020), Giannitsarou et al. (2021); Goenka et al. (2020, 2021), and Jones et al. (2020) for a partial list.

[14] See for example Acemoglu et al. (2021), Alvarez et al. (2020), Eichenbaum et al. (2021); Giannitsarou et al. (2021), and Jones et al. (2020).

not model capital accumulation.[15] Some of them model flow utility in different health states as constant.[16] The evidence, however, is that consumption has fallen faster and in larger magnitude during the pandemic than in the 2008 Financial Crisis (McKinsey & Co 2021). The effect on investment is of a similar magnitude as consumption and is expected to persist for a longer period even when the expectation is that the pandemic is going to end (Bloom et al. 2020). Thus, understanding how consumption and investment behave if the disease is endemic is important to understand the full impact on economic outcomes. In our model, we see that capital, output and consumption decrease relative to the disease free steady state. This is due to several channels: through impacting labor supply and via disease mortality which changes population size effectively endogenizing discounting which impacts intertemporal choices. The change in effective discounting not only affects the consumption-saving decision but also how current welfare changes are weighed relative to future ones. The effect of disease incidence and mortality can induce a policy response of quarantines which further impacts consumption. Treating the flow utility from consumption (and investment) as constant will underestimate the effect of the disease as it would capture only the changes in the fractions of population in the different health states but not changes in consumption.

It is already well-known in the literature that epidemiology dynamics are not convex and the first order conditions to control problems need not be sufficient. With disease related mortality, as population size changes with the level of infection, effectively the discount rate becomes endogenous. We establish the appropriate transversality and sufficiency conditions for the $SIRS$ model using a direct argument.[17]

The plan of the paper is as follows: Sect. 2 introduces the $SIRS$ model, Sect. 3 the economic epidemiology model, Sect. 4 studies the equilibrium steady states, Sect. 5 the comparative statics of equilibrium steady state outcomes, Sect. 6 the transversality and sufficiency conditions, and Sect. 7 concludes.

## 2 The SIRS epidemiology model

For the epidemiology dynamics we use a $SIRS$ model with standard incidence, homogeneous mixing, and disease related mortality. An individual can be in one of three health states, $S$, where the individual is healthy and susceptible to the disease, $I$ where the individual is infective that is infected and infectious enough to transmit the disease, or $R$ where the individual is recovered and has immunity to the disease.

Figure 1 is the transfer diagram for the epidemiology model. The parameters in the model are $b$ the birth rate, i.e. new flow of susceptibles into the population which includes birth, travel, migration, etc., $d$ the death or death or exit rate of individuals

[15] See Acemoglu et al. (2021), Alvarez et al. (2020), Eichenbaum et al. (2021), Giannitsarou et al. (2021), Jones et al. (2020) for a partial list. Aspri et al. (2021), and Eichenbaum et al. (2020) include capital and focus on the short-run. Goenka et al. (2020, 2021) include capital and are closest to this paper but use $SIS$ and $SIR$ dynamics respectively.

[16] e.g. Acemoglu et al. (2021), Alvarez et al. (2020), Giannitsarou et al. (2021)

[17] Goenka et al. (2014) show this for the $SIS$ model without mortality, Goenka et al. (2020) show this for the $SIS$ model with mortality, and Goenka et al. (2021) show this for the $SIR$ model with mortality.

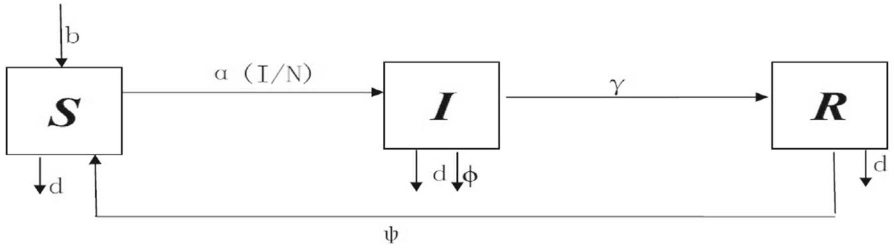

**Fig. 1** The Transfer Diagram For the $SIRS$ Epidemiology Model with disease related mortality. Note: In a $SIRS$ epidemiology model, the population is divided into three groups: the susceptible denoted as $S$, the infected denoted as $I$, and the recovered $R$. The birth rate is $b$ and newborns are born healthy and susceptible. All individuals irrespective of health status die at the rate $d$. The susceptible get infected at the rate $\alpha \frac{I}{N}$, the infected recover at the rate $\gamma$ and might die at the rate $\phi$ as a result of being infected. The recovered may lose immunity at the rate $\psi$

which is not related to the infectious disease, $\alpha$ is the contact rate of adequate contacts that can transmit the disease, $\gamma$ is the recovery rate from the disease, $\phi$ is the mortality from infections due to the disease, and $\psi$ is the rate at which the recovered lose immunity. The $SIR$ dynamics where there is no escape from immunity is a special case of the $SIRS$ dynamics with $\psi = 0$. We use the standard or density dependence model where the transmission of the disease depends on the fraction of infected rather than number of infected. In the mass action model, there are scale effects which are seen in herd models but are thought not to characterize human interactions where the pattern of interactions is relatively invariant to population size.

In this paper we concentrate on the control of the disease through the imposition of a lockdown. This is motivated by Covid-19[18] for which there were no proven prophylactic medicines or proven treatments for recovery at the time of writing the paper. Thus, we treat the epidemiological parameters $\alpha$, $\gamma$, $\psi$ as fixed.[19] All methods of control considered in the paper are non-pharmaceutical interventions (NPIs).[20] As this analysis is motivated by lockdowns as a method of disease control imposed by governments (or optimal self-imposed self-isolation) when there are no medical interventions, we concentrate on the socially optimal solution abstracting away from these issue which have already been studied in our earlier work. We also do not model the disease externality in this paper as Goenka and Liu (2020) has a detailed analysis of it in an general equilibrium framework $SIS$ framework.[21]

The way we model lockdown is that a fraction, $\theta$, with $0 \leq \theta \leq 1$, of both healthy (Susceptible and Recovered) and the infected (Infective) population is quarantined. There is homogeneous mixing and no effective track-and-trace-and-isolate (TTI) program that will isolate the infective and quarantine only those who have been exposed

[18] As well as other coronaviruses including SARS and MERS.

[19] In earlier papers, Goenka et al. (2014, 2021), Goenka and Liu (2012, 2020) and Rowthorn and Toxvaerd (2020) $\alpha$ and $\gamma$ are endogenized.

[20] In the paper we do not model vaccinations which introduce additional epidemiology states and a different set of issues. There is also considerable diversity across countries on which vaccines are deployed and the extent of coverage in the population.

[21] See also Toxvaerd (2019, 2020) which also studies this externality in a partial equilibrium $SIS$ model.

to infection.[22] The experience of quarantines shows that they have been successful in varying degrees as there are issues of both compliance and enforcement. We model the effectiveness of the quarantine or compliance with the lockdown to reduce infections by the parameter $\zeta$ , with $0 \leq \zeta \leq 1$. When $\zeta = 0$ the lockdown is not effective and with $\zeta = 1$ it is fully effective. In the paper we concentrate on partial effectiveness of lockdowns, $0 < \zeta < 1$. The determinants of compliance with a lockdown are many with complex interactions between them.[23] In this paper we treat it as a parameter.

The $SIRS$ epidemiology model with quarantines is given by the following system of differential equations:

$$\begin{aligned}
\dot{S} &= bN - \frac{\alpha S(1-\zeta\theta)I(1-\zeta\theta)}{N} - dS + \psi R \\
\dot{I} &= \frac{\alpha S(1-\zeta\theta)I(1-\zeta\theta)}{N} - \gamma I - dI - \phi I \\
\dot{R} &= \gamma I - dR - \psi R \\
\dot{N} &= (b-d)N - \phi I
\end{aligned}$$

Since $N = S + I + R$, we define $s = S/N$ and $i = I/N$. The proportion of the recovered $r = R/N = 1 - s - i$. Defining $\dot{i} \equiv \Omega$, the $SIRS$ epidemiological model can be reduced to:

$$\dot{s} = b - bs - \alpha(1-\zeta\theta)^2 si + \psi(1-s-i) + \phi si \quad (1)$$
$$\Omega = \alpha(1-\zeta\theta)^2 si - bi - \phi i - \gamma i + \phi i^2, \quad (2)$$

where the total population grows at the rate $b - d - \phi i$. Note that the population growth rate here is endogenous and affected by the prevalence of infectious diseases. We denote the steady state of a variable $x$ as $x^\infty$ to distinguish it from the optimal value in a trajectory in the later part of the paper which is denoted as $x^*$.

**Proposition 1** *(Busenberg and van den Driessche (1990), Mena-Lorca and Hethcote (1992)) Consider the epidemiological model given by equation (1) - (2). Then*

1. *The disease free steady state with $s^\infty = 1$, $i^\infty = 0$ and $r^\infty = 0$ always exists. It is stable when $\frac{\alpha(1-\zeta\theta)^2}{b+\gamma+\phi} \leq 1$, and unstable when $\frac{\alpha(1-\zeta\theta)^2}{b+\gamma+\phi} > 1$.*
2. *When $\frac{\alpha(1-\zeta\theta)^2}{b+\gamma+\phi} > 1$, there exists a unique endemic steady state with $0 < s^\infty < 1$, $0 < i^\infty < 1$ and $0 < r^\infty < 1$, which is stable. The endemic steady state $(s^\infty, i^\infty)$*

[22] There is diversity across countries on the effectiveness of TTI programs. Many countries do have test-and-track programs for Covid (e.g. Denmark, Singapore, Japan, Korea, Germany, China, Vietnam) and many of the countries that have had the largest number of infections do not have fully effective ones (e.g. US, UK, India, Brazil, Sweden, Russia, South Africa). Even with test-and-tracking, whether the infected and potentially infected can be isolated varies considerably and depends on personal compliance.

[23] The emerging literature on the determinants of compliance shows that some of the factors are trust of policy makers (Bargain and Aminjonov 2020; Vinck et al. 2019), civic engagement (Barrios et al. 2021), age (Belot et al. 2021), social capital (Borgonovi et al. 2020; Mazzonna 2020), political views (Brodeur et al. 2021), broader socio-economic determinants including gender, political partisanship and risk tolerance (Fan et al. 2020),(Papageorge et al. 2020).

*is the solution to the following system of equations:*

$$\frac{\alpha(1-\zeta\theta)^2}{\phi}\left(\alpha(1-\zeta\theta)^2-\phi\right)s^2$$
$$+\left(\phi+\gamma-\psi-\frac{\phi+b+\gamma}{\phi}\alpha(1-\zeta\theta)^2+\frac{\alpha(1-\zeta\theta)^2}{\phi}\psi\right)s$$
$$+b+\psi-\frac{\phi+b+\gamma}{\phi}\psi=0 \tag{3}$$
$$i=\frac{\phi+b+\gamma}{\phi}-\frac{\alpha(1-\zeta\theta)^2}{\phi}s \tag{4}$$

The papers by Busenberg and van den Driessche (1990) and Mena-Lorca and Hethcote (1992) did not have quarantines. We can easily amend their result by noting how it affects the contact rate. Note that even though the fraction of susceptibles, $s$, is given by a quadratic equation there is only one admissible solution in the range $0 < s < 1$ in the pure epidemiology model (see Busenberg and van den Driessche (1990)).

The reproduction number, $R_0 = \dfrac{\alpha(1-\zeta\theta)^2}{b+\gamma+\phi}$, which is number infections generated by an infected individual plays a key role in the evolution of the disease. Proposition 1 indicates that the disease is endemic only when $R_0 > 1$. In the pure epidemiology model there is a cutoff lockdown level $\bar{\theta}$ such that $R_0 = \dfrac{\alpha(1-\zeta\bar{\theta})^2}{b+\gamma+\phi} = 1$. Thus, given the parameters in the pure epidemiology model a lockdown above $\bar{\theta}$ does not need to be imposed to eradicate the disease.[24]

## 3 The economic epidemiology model

The model is based on the economic epidemiology model in Goenka and Liu (2012, 2020) and Goenka et al. (2014, 2020, 2021) but with $SIRS$ disease dynamics to include disease related mortality and to model quarantines. To avoid keeping track of the cross-sectional distribution of the healthy and infected individuals, and to stay close to the canonical growth model, we adopt the framework of a large representative household.

### 3.1 The model

*Households* We assume the economy is populated by a continuum of non-atomic identical households who are representative decision-making agents. In the absence of the disease, the size of the population in each household grows at the rate of $b-d \geq 0$, where $b$ is the birth rate and $d$ is the death rate. Within each household, an individual is either healthy, infected, or recovered from the disease.

[24] This is also noted in Giannitsarou et al. (2021).

We model the infectious disease as having two effects-morbidity, i.e. illness that reduces productivity of the infected, and disease related mortality. We make the simplifying assumption that an infected individual is incapacitated by the disease or that the productivity falls to zero.[25] For Covid-19 many of the infected are asymptomatic and to the extent they are not isolated, their productivity is not affected by infections.This could be modelled by introducing the health state of Exposed, $E$. This is important for short-run dynamics and for modelling some interventions but given our focus in this paper we do not model it. Even for "mild" cases that do not require hospitalization, the effect of the disease is debilitating and can have long lasting tail effects, i.e. Long Covid (see Chertow et al. (2021); Nalbandian et al. (2021), and Sigfrid et al. (2021)). UK data shows that 1/3 of those admitted to hospitals for acute Covid are readmitted in five months, and 1/10 die after discharge. We assume the labor is supplied inelastically.[26] If $i$ is the fraction of household infected, a proportion $\phi$ of these succumb to the disease.

We study simplest model where the only way to control infection, and hence, disease related mortality is through lockdowns or quarantines denoted by $\theta$. Individuals who are not infected and not quarantined can participate in the labor market with productivity equal to 1 so that their labor supply is $(1-\theta)(S+R)$. People who are healthy but quarantined can work at home with productivity $\xi$ (with $0 \leq \xi \leq 1$), so the labor supply of healthy quarantined at home is $\xi\theta(S+R)$). Some individuals are in occupations where they cannot work from home. The emerging evidence is that there is considerable heterogeneity across occupations and characteristics of individuals who work from home on the loss of productivity.[27] When $\xi = 0$ the productivity of working from home is zero. In this case, full lockdown ($\theta = 1$) is never desirable as the total output would be zero.[28] With $\xi = 1$ working at home does not affect productivity and full lockdown is always the best choice and the economy will be in a disease free steady state. In the paper, we focus on the case where $0 < \xi < 1$.

The total effective labor force is

$$L = (1-\theta+\xi\theta)(S+R).$$

As a fraction of the population this is:

$$l = L/N = (1-\theta+\xi\theta)(1-i) \tag{5}$$

[25] Jo et al. (2020) estimate both YLL and YLD in South Korea, and Nurchis et al. (2020) for Italy.

[26] In Goenka and Liu (2012) we endogenize the labor-leisure choice with $SIS$ disease dynamics and show that the dynamics are invariant under standard assumptions.

[27] See Adams-Prassl et al. (2021) Alipour et al. (2020); Bartik et al. (2020); Dingell and Neiman (2020); Gottlieb et al. (2021), and Hensvik et al. (2020)) for some examples of this emerging literature. The effect on productivity is affected by occupation and industry, number and age of children, care responsibilities, gender issues, access to technology, amongst other things. Soares et al. (2021) show those working from home may have different wage profiles reflecting differing productivity across countries.

[28] An Inada condition on the utility function, $\lim_{c\to 0} u'(c) \to \infty$ will ensure this. For the rest of the analysis we do not need such a condition. Without an Inada condition there could be situations in the transitional dynamics where there is complete lockdown if there is sufficiently high mortality from the disease.

*Production* The production side of the model is a standard neo-classical growth model where households can invest in capital which is productive next period and depreciates at rate $\delta$.[29] Households own representative firms that use capital and labor as inputs. The production function $f(k, l)$ is a neo-classical production function, i.e. concave, with positive marginal products, homogeneous of degree 1, satisfies the Inada conditions, and is twice-continuously differentiable. The depreciation rate of capital is denoted by $\delta \in (0, 1]$.

The law of motion for physical capital accumulation is standard:

$$\dot{k} = f(k, l) - c - \delta k - (b - d - \phi i)k \tag{6}$$

### 3.2 The welfare function and planning problem

The objective function is the discounted sum of utility from consumption minus the welfare loss from infections, multiplied by the size of the population. This is an extension of the standard utilitarian welfare function which is based only on utility from consumption to incorporate the loss from infections. It assumes that each household is weighted equally and there is perfect insurance within each household. Multiplying the household's extended welfare by the population size is standard in the literature and will capture the effect of variation in the population size due to disease related mortality. Thus, the welfare function to be maximized is:

$$\int_0^\infty e^{-\rho t}[u(c) - \chi \nu(i)]N dt$$
$$= \int_0^\infty e^{-\int_0^t (\rho - b + d + \phi i_\tau) d\tau}[u(c) - \chi \nu(i)]N_0 dt$$

where $\rho$ is the discount rate with $\rho > b - d$ , $\nu(i)$ is the welfare loss from infections, and $\chi$ is the weight given to loss from infections. Different societies/planners may assign different loss from an additional infection which is given by the shape of $\nu$ as well the relative weight of utility of consumption and disutility from infections which is captured by $\chi$. The specification allows for different effects from infections. For example, $\nu(i)$ could be of the form $\nu(i) = \kappa(i) + \omega(\phi i)$ so that the losses from infection given by $\kappa(\cdot)$ and mortality given by $\omega(\cdot)$ are treated differently. In this paper we focus on the case of $\nu(\phi i)$ so that the only disease mortality affects welfare directly. We model the welfare loss from infections as separable from consumption as we do not want to suggest that loss from infections and mortality is substitutable with consumption. These losses are likely to have differential effect on different income groups or economies and we think they primarily come from the budget constraint rather than how different groups view infections and losses.

29 Goenka and Liu (2020) have an endogenous growth model where there is human capital accumulation and households choose time to work and time for human capital accumulation. It uses $SIS$ dynamics without disease related mortality.

**Assumption 1** The welfare function:

1. $u(c) : \mathbb{R}_+ \to \mathbb{R}$ is $\mathcal{C}^2$ with $u' > 0$ and $u'' < 0$;
2. $\nu(i) : \mathbb{R}_+ \to \mathbb{R}$ is a convex function with $\nu' > 0$ and $\nu'' \geq 0$ and $\nu(0) = 0$
3. The disutility weight $\chi \geq 0$.

When there is no disease prevalence, the disutility from disease mortality is of course zero. For the case of loss from mortality only, we can write it as $\nu(\phi i)$ which is equivalent to the objective as $\phi$ is a constant in this paper. In the paper, we concentrate on this case but the framework allows welfare loss from infections which may become important as mortality is controlled but the effects of Long Covid remain. When $\chi = 0$, the model becomes the standard model where no weight is given to loss in welfare to disease and infections. How to weight the loss from mortality is an important one and we discuss this in further detail in Sect. 5.2.

For rest of the paper we will specialize the welfare function to make $\nu$ a function of disease related mortality so as to be closer to the existing literature. Note, that the economic loss due to infection is already incorporated in the constraints and the loss due to the change in population size is coming from the fact that we evaluate total rather than per capita utility.

The objective function becomes:

$$\max_{\{c,\theta\}} \int_0^\infty e^{-\rho t}[u(c) - \chi \nu(\phi i)] N dt$$
$$= \int_0^\infty e^{-\int_0^t (\rho - b + d + \phi i_\tau) d\tau}[u(c) - \chi \nu(\phi i)] N_0 dt$$

where $\nu(\phi i)$ is the loss in welfare from disease mortality with weight $\chi$.

As the population size is varying, the discount factor is endogenous and varies with infections in the population To solve this maximization problem with the endogenous discount rate, we define the following variable which is the effective discount rate (see Uzawa 1968),

$$\Lambda_t = \int_0^t (\rho - b + d + \phi i_\tau) d\tau,$$

where

$$\dot{\Lambda}_t = \rho - b + d + \phi i_t. \tag{7}$$

The inter-temporal welfare function is thus:

$$\max_{\{c,\theta\}} \int_0^\infty e^{-\Lambda} [u(c) - \chi \nu(\phi i)] N_0 \, dt. \tag{8}$$

Note that with changes in infections, $i$, disease related mortality, $\phi i$, changes the effective discount rate and thus, $\Lambda$ is affected by a state variable.

## 4 Characterization of steady states

The social planner problem is to maximize (8) (we suppress the time subscript) subject to equations (1),(2),(5),(6) and (7) with $0 \leq \theta \leq 1,\ s \leq 1$ and $i \geq 0$.

As the effective discount rate varies with the rate of infections (which are not monotonic) there can be a time-consistency problem. To avoid this we work the present value Hamiltonian with the additional state variable of the changing discount rate:

$$\begin{aligned} H = \ & e^{-\Lambda}[u(c) - \chi\nu(\phi i)] + \lambda_1\{f(k, (1-\theta+\xi\theta) \\ & (1-i)) - c - \delta k - (b-d-\phi i)k\} \\ & +\lambda_2\{b - bs - \alpha(1-\zeta\theta)^2 si + \psi(1-s-i) + \phi si\} \\ & +\lambda_3\{\alpha(1-\zeta\theta)^2 si - bi - \gamma i - \phi i + \phi i^2\} \\ & +\lambda_4\{\rho - b + d + \phi i\} + \mu_1\theta + \mu_2(1-\theta) + \mu_3(1-s) + \mu_4 i, \end{aligned} \tag{9}$$

where $\lambda_1, \ldots, \lambda_4$ are costate variables and $\mu_1, \ldots, \mu_4$ are Lagrange multipliers.

The Hamiltonian is not jointly concave in the state and control variables so none of the existing sufficiency conditions apply.

***Remark 1*** The Hamiltonian is not jointly concave in state and control variables. In particular, the condition for the Hessian matrix to be semi-negative definite which requires the principal minors $M_j (j = 1, ..., 6)$ alternate in sign, starting with a negative determinant is not satisfied in our model if the welfare function is positive, i.e. if $u(c) - \chi\nu(\phi i) > 0$.

Let us rewrite the Hamiltonian as $H(k, i, \Lambda, s, c, \theta)$. Then it is easy to check the first minor $M_1 = |H_{kk}| = \lambda_1 f_{11} < 0$. The second minor is $M_2 = \begin{vmatrix} H_{kk} & H_{ki} \\ H_{ik} & H_{ii} \end{vmatrix}$. We alsohave

$$\begin{aligned} M_3 &= \begin{vmatrix} H_{kk} & H_{ki} & 0 \\ H_{ik} & H_{ii} & H_{i\Lambda} \\ 0 & H_{\Lambda i} & H_{\Lambda\Lambda} \end{vmatrix} = H_{\Lambda\Lambda}M_2 + (-1)^{2+3}H_{\Lambda i}\begin{vmatrix} H_{kk} & 0 \\ 0 & H_{\Lambda\Lambda} \end{vmatrix} \\ &= H_{\Lambda\Lambda}(M_2 - H_{\Lambda i}H_{kk}). \end{aligned}$$

Because $H_{\Lambda\Lambda} = e^{-\Lambda}[u(c) - \chi\nu(\phi i)] > 0$, $H_{\Lambda i} = e^{-\Lambda}\chi\phi\nu'(\phi i)] > 0$, $H_{kk} < 0$, we have

$$M_3 = H_{\Lambda\Lambda}(M_2 - H_{\Lambda i}H_{kk}) > 0 \text{ if } M_2 > 0.$$

So the condition for the Hessian being semi-negative fails. □

In Sect. 6 we directly establish the appropriate transversality conditions and the sufficiency of the first order conditions using a direct argument following Leitmann and Stalford (1971).

The necessary and sufficient first order conditions are:

$$c : e^{-\Lambda}u'(c) = \lambda_1 \tag{10}$$

$$\theta : \mu_2 - \mu_1 = 2(1-\zeta\theta)\zeta\alpha si(\lambda_2 - \lambda_3) - \lambda_1 f_2(k,l)(1-\xi)(1-i) \quad (11)$$
$$k : \dot{\lambda_1} = -\lambda_1[f_1(k,l) - \delta - b + d + \phi i] \quad (12)$$
$$s : \dot{\lambda_2} = -\lambda_2[-b - \alpha(1-\zeta\theta)^2 i - \psi + \phi i] - \lambda_3\alpha(1-\zeta\theta)^2 i + \mu_3 \quad (13)$$
$$\begin{aligned} i : \dot{\lambda_3} = {} & e^{-\Lambda}\chi v'(\phi i)\phi + \lambda_1[f_2(k,l)(1-\theta+\xi\theta) - \phi k] \\ & +\lambda_2[\alpha(1-\zeta\theta)^2 s + \psi - \phi s] \\ & -\lambda_3[\alpha(1-\zeta\theta)^2 s - b - \gamma - \phi + 2\phi i] - \lambda_4\phi - \mu_4 \end{aligned} \quad (14)$$
$$\Lambda : \dot{\lambda_4} = e^{-\Lambda}[u(c) - \chi v(\phi i)] \quad (15)$$
$$\mu_1 \geq 0, \quad \theta \geq 0, \quad \mu_1\theta = 0 \quad (16)$$
$$\mu_2 \geq 0, \quad 1-\theta \geq 0, \quad \mu_2(1-\theta) = 0 \quad (17)$$
$$\mu_3 \geq 0, \quad s \leq 1, \quad \mu_3(1-s) = 0 \quad (18)$$
$$\mu_4 \geq 0, \quad i \geq 0, \quad \mu_4 i = 0 \quad (19)$$

**Proposition 2** *There always exists a unique disease free steady state with* $s^\infty = 1, i^\infty = 0$*,* $\theta^\infty = 0$*,* $l^\infty = 1$ *and* $k^\infty$ *and* $c^\infty$ *are determined by*

$$\begin{aligned} f_1(k,1) &= \rho + \delta \\ c &= f(k,1) - \delta k - (b-d)k. \end{aligned}$$

***Proof*** From $\dot{s} = 0$ and $\Omega = 0$, we have one disease free steady state $s^\infty = 1, i^\infty = 0$ and thus $\mu_3 > 0, \mu_4 > 0$. From equation (11), we have

$$\mu_1 - \mu_2 = \lambda_1 f_2(k,l)(1-\xi).$$

If $\xi < 1$, then $\mu_1 > \mu_2 \geq 0$. Therefore, $\mu_1$ is strictly positive and implies $\theta^\infty = 0$. Then, from equation (5), $l^\infty = 1$. From equation (12), we have

$$\frac{\dot{\lambda_1}}{\lambda_1} = -[f_1(k,1) - \delta - b + d].$$

Moreover, from equation (10), we have

$$\frac{\dot{\lambda_1}}{\lambda_1} = -(\rho - b + d) + \frac{u''(c)}{u'(c)}\dot{c}.$$

Since the economy is bounded, all economic variables including $k$, $c$ and $l$ are constant in the steady state. That is, $\dot{c} = 0$ in the steady state. Thus, combing the above two equations, we have

$$f_1(k,l) = \rho + \delta,$$

from which we can solve for $k^\infty$. $c^\infty$ is derived from equation (6) with $\dot{k} = 0$ in the steady state. □

When the contact rate without any lockdown is small enough such that the reproduction number $R_0 = \frac{\alpha}{b+\gamma+\phi} < 1$, the disease is eradicated in the long-run. The economy is the same as a standard neo-classical economy without any disease prevalence. The physical capital, total output and consumption in the disease free steady state provide the benchmark for us to evaluate the economic variables in a disease endemic steady state later. Note that when $R_0 = \frac{\alpha}{b+\gamma+\phi} > 1$ there also exists a disease endemic steady state. The disease free steady state is unstable and the disease endemic steady state is stable (see Appendix A.1).

Next, we look at the disease endemic steady state, which exists only when $R_0 > 1$. Note that in the economic epidemiology model, $R_0$ is endogenous as it depends on the optimal level of quarantine, $\theta$. Thus, the key variable to determine in the disease endemic steady state is $\theta \in [0, \bar{\theta})$. We start by defining a function $G$-the net marginal benefit of implementing lockdown $\theta$.

The marginal benefit of implementing a lockdown is in controlling the disease prevalence by reducing the effective contact rate. When we increase the lockdown measure $\theta$, the effective contact rate reduces by $2(1-\zeta\theta)\zeta\alpha$. This implies the fraction of $2(1-\zeta\theta)\zeta\alpha si$ less of the susceptible become infected. That is, the number of the susceptible increases by $2(1-\zeta\theta)\zeta\alpha si$ and the number of the infected decreases by $2(1-\zeta\theta)\zeta\alpha si$. So the effect of increasing the lockdown measure on the total utility of the household is $2(1-\zeta\theta)\zeta\alpha si(\lambda_2-\lambda_3)$, as the shadow value of the susceptible and the infected are $\lambda_2$ and $\lambda_3$, respectively. The difference between the two captures the effect of quarantine on the evolution of infections. Therefore, the marginal benefit of increasing the lockdown measure $\theta$ is $2(1-\zeta\theta)\zeta\alpha si(\lambda_2-\lambda_3)$.

The marginal cost of implementing a lockdown is the reduction in labor supply, as we assume that the effective labor supply is $l = (1-\theta+\xi\theta)(1-i)$. For one unit of increase in lockdown measure $\theta$, the labor supply decreases by $(1-\xi)(1-i)$ and the output decreases by $f_2(k,l)(1-\xi)(1-i)$. Therefore, the marginal cost of the lockdown is $f_2(k,l)(1-\xi)(1-i)\lambda_1$, as the shadow value of the output is $\lambda_1$.

Thus, the net marginal benefit of implementing lockdown measure $\theta$ is:

$$2(1-\zeta\theta)\zeta\alpha si(\lambda_2-\lambda_3) - f_2(k,l)(1-\xi)(1-i)\lambda_1.$$

Moreover, we have $\lambda_1 = e^{-\Lambda}u'(c)$, $\tilde{\lambda}_2 = \lambda_2/e^{-\Lambda}$ and $\tilde{\lambda}_3 = \lambda_3/e^{-\Lambda}$. Thus, changes in disease related mortality by affecting the effective discount rate affect the shadow price of the three different health states. If we look at the expression for the net marginal benefit of implementing a lockdown we see that the shadow price of capital enters negatively. As utility function is concave, lower consumption levels will imply lower net marginal benefits. This can be seen in the experience as low and middle income countries have generally had smaller quarantines during Covid. This also matches the microeconomic evidence that lower income groups typically had smaller declines in mobility during lockdowns (See for e.g. Bonaccorsi et al. (2020); Coven and Gupta (2020); Weill et al. (2020)). While marginal product of labor also enters negatively, its effects are complex as the effective labor force depends not only on disease dynamics, but also on quarantines, and productivity of working from home.

**Definition 1** Define the net marginal benefit of imposing a lockdown, $G$:

$$G(\theta) = 2(1-\zeta\theta)\zeta\alpha si(\tilde{\lambda}_2 - \tilde{\lambda}_3) - \tilde{\lambda}_1 f_2(k,l)(1-\xi)(1-i), \tag{20}$$

where

$$\frac{\alpha(1-\zeta\theta)^2}{\phi}\left(\alpha(1-\zeta\theta)^2 - \phi\right)s^2 + \left(\phi + \gamma - \psi - \frac{\phi+b+\gamma}{\phi}\alpha(1-\zeta\theta)^2 + \frac{\alpha(1-\zeta\theta)^2}{\phi}\psi\right)s + +b + \psi - \frac{\phi+b+\gamma}{\phi}\psi = 0 \tag{21}$$

$$i = \frac{\phi+b+\gamma}{\phi} - \frac{\alpha(1-\zeta\theta)^2}{\phi}s \tag{22}$$

$$l = (1-\theta+\xi\theta)(1-i) \tag{23}$$

$$f_1(k,l) = \rho + \delta \tag{24}$$

$$f(k,l) = c + \delta k + (b - d - \phi i)k \tag{25}$$

$$M = -\chi v'(\phi i)\phi - \frac{u(c) - \chi v(\phi i)}{\rho - b + d + \phi i}\phi - u'(c)[f_2(k,l)(1-\theta+\xi\theta) - \phi k] \tag{26}$$

$$\tilde{\lambda}_2 = \lambda_2/e^{-\Lambda} = \frac{\alpha(1-\zeta\theta)^2 iM}{(\rho+d+\psi+\alpha(1-\zeta\theta)^2 i)(\rho+d+\gamma+\phi-\phi i - \alpha(1-\zeta\theta)^2 s) + \alpha(1-\zeta\theta)^2 i(\alpha(1-\zeta\theta)^2 s + \psi - \phi s)} \tag{27}$$

The economic epidemiological model is a combination of the epidemiological model and the economic model. The connection between the two models is the lockdown measure $\theta$ which is determined by the economic costs and benefits. The evolution of the disease depends on the lockdown measure $\theta$, along with other epidemiological parameters. Once we determine the optimal $\theta$ that satisfies the equilibrium conditions, the $SIRS$ epidemiological model-equation (21) and (22) determine the steady state $s^{\infty}$ and $i^{\infty}$. Then, from the economic component of the model, once we know the disease prevalence we can determined the labor force (equation (23)), physical capital (equation (24)) and consumption (equation (26)). Equation (26) and (27) provide the present shadow value of the susceptible and the infected, which are used to calculate the marginal benefit of controlling. the disease.Therefore, by equations (21)–(27), all variables in the model are functions of $\theta$. Thus, the function $G$ is essentially a function of the lockdown choice $\theta$. In other words, once level of lockdown in the model is pinned down in equilibrium, we can solve for all the other variables.

**Proposition 3** *There are three scenarios:*[30]

- *If* $G(\theta)|_{\theta=0} < 0$, *then* $\theta^{\infty} = 0$;
- *If* $G(\theta)|_{\theta=1} > 0$, *then* $\theta^{\infty} = 1$;

[30] We could have used two superscripts $\infty$ and $*$ to emphasize that this is the optimal value of the variable in a steady state but we suppress the second one for notational brevity.

- *Otherwise,* $\theta^{\infty}$ *is determined by* $G(\theta^{\infty}) = 0$.

*Given the optimal* $\theta^{\infty}$*, an endemic steady state exists if* $\frac{\alpha(1-\zeta\theta^{\infty})^2}{b+\gamma+\phi} > 1$.

*Given the optimal* $\theta^{\infty}$*, the steady state variables* $s^{\infty}$*,* $i^{\infty}$*,* $l^{\infty}$*,* $k^{\infty}$ *and* $c^{\infty}$ *are determined by equations (21)–(27).*

***Proof*** From $\dot{s} = 0$ and $\Omega = 0$, we have one endemic steady state with $s^{\infty}$ and $i^{\infty}$ given by equations (21) and (22). We can see that $s^{\infty}$ and $i^{\infty}$ are functions of the lockdown $\theta$. The steady state exists only if $\frac{\alpha(1-\zeta\theta^{\infty})^2}{b+\gamma+\phi} > 1$. Then, we have $\mu_3 = 0$ and $\mu_4 = 0$.

From equation (5), $l^{\infty} = (1-\theta+\xi\theta)(1-i^{\infty})$. From equation (12), we have

$$\frac{\dot{\lambda}_1}{\lambda_1} = -[f_1(k,l) - \delta - b + d + \phi i].$$

Moreover, from equation (10), we have

$$\frac{\dot{\lambda}_1}{\lambda_1} = -(\rho - b + d + \phi i) + \frac{u''(c)}{u'(c)}\dot{c}.$$

Since the economy is bounded, all economic variables including $k$, $c$ and $l$ are constant in the steady state. That is, $\dot{c} = 0$ in the steady state. Thus, combing the above two equations, we have

$$f_1(k,l) = \rho + \delta,$$

from which we can solve for $k^{\infty}$ and $c^{\infty}$ is derived from equation (6) with $\dot{k} = 0$ in the steady state.

All co-state variables $\lambda_1$, $\lambda_2$ and $\lambda_3$ grow at the same rate:

$$g = \frac{\dot{\lambda}_1}{\lambda_1} = \frac{\dot{\lambda}_2}{\lambda_2} = \frac{\dot{\lambda}_3}{\lambda_3} = -(\rho - b + d + \phi i)$$

Since $g = \frac{\dot{\lambda}_4}{\lambda_4} = \frac{e^{-\Lambda}[u(c)-\chi\nu(\phi i)]}{\lambda_4}$, we have

$$\lambda_4 = \frac{e^{-\Lambda}[u(c) - \chi\nu(\phi i)]}{g}.$$

Define $\tilde{\lambda}_2 = \lambda_2/e^{-\Lambda}$ and $\tilde{\lambda}_3 = \lambda_3/e^{-\Lambda}$. From equation (13) and (14), we have

$$\begin{aligned}
&[\rho + d + \psi + \alpha(1-\zeta\theta)^2 i]\tilde{\lambda}_2 - \alpha(1-\zeta\theta)^2 i\tilde{\lambda}_3 = 0\\
&(\alpha(1-\zeta\theta)^2 s + \psi - \phi s)\tilde{\lambda}_2 + [\rho + d + \gamma + \phi - \phi i - \alpha(1-\zeta\theta)^2 s]\tilde{\lambda}_3 =\\
&= -\chi\nu'(\phi i)\phi - \frac{u(c) - \chi\nu(\phi i)}{\rho - b + d + \phi i}\phi - u'(c)[f_2(k,l)(1-\theta+\xi\theta) - \phi k]
\end{aligned}$$

Then, we can solve for $\tilde{\lambda}_2$ and $\tilde{\lambda}_3$.

Thus, from equation (11), we have

$$\begin{aligned}\mu_2 - \mu_1 &= 2(1-\zeta\theta)\zeta\alpha s i(\lambda_2 - \lambda_3) - \lambda_1 f_2(k,l)(1-\xi)(1-i) \\ &= e^{-\Lambda}\, G(\theta),\end{aligned}$$

Moreover, equations (16) and (17) imply:

1) If $G(\theta) < 0$ when $\theta = 0$, that is, the marginal benefit of lockdown is smaller than the marginal cost, the endemic steady state is one with no lockdown ($\theta^\infty = 0$).
2) If $G(\theta) > 0$ when $\theta = 1$, that is, the marginal benefit of lockdown is larger than the marginal cost, the endemic steady state is one with full lockdown ($\theta^\infty = 1$);
3) Otherwise, the endemic steady state is one with partial lockdown ($0 < \theta^\infty < 1$), where $\theta^\infty$ is determined by solving $G(\theta^\infty) = 0$. □

## 5 Simulations

There are four sets of parameters to be determined-the epidemiological, relating to lockdowns, demographic, and economic parameters. We use a quarterly frequency in our model simulation. The economic and demographic parameters are standard in the literature. Thus, we first focus on the parameters of the $SIRS$ epidemiological model-the contact rate $\alpha$, the recovery rate $\gamma$, the rate of losing immunity $\psi$ and the disease related mortality rate $\phi$, and those relating to lockdowns-the efficacy of lockdowns, $\zeta$, the productivity of working from home, $\xi$, and the weight on welfare loss from disease related mortality, $\chi$. We examine how the choices of some of these parameters affect the long-run prediction of the $SIRS$ epidemiological model.

### 5.1 The epidemiological parameters and simulation of the SIRS model

The papers on epidemiology of Covid often use the daily data, and focus on estimation of the two parameters-the recovery rate $\gamma$ and the contact rate $\alpha$, which are shown to lie in a wide range. This is partly due to the differences in modelling choices, data selection, and demographics of the sample group. Bertozzi et al. (2020) have fitted the confirmed case and mortality data from three US states (California, New York, and Indiana) to the $SIR$ model using maximum likelihood estimation. The $SIR$ model, where the recovered will not lose immunity, is a special case of the $SIRS$ model. Their estimates on the recovery rate $\gamma$ lie in the range of $0.06 - 0.19$, implying that it takes $5 - 17$ days to recover. Their estimates of the basic reproduction number $R_0$ lie in the range of $2.1 - 4.4$, which implies the estimates of the contact rate are in the range of $0.26 - 0.41$, that is, a generation time of $2.4 - 3.8$ days.[31] The generation

[31] For California, the estimates on the recovery rate $\gamma$ and $R$ are 0.14 and 2.4 if fitted to confirmed cases, and 0.12 and 2.7 if fitted to mortality data. For Indiana, the estimates on the recovery rate $\gamma$ and $R$ are 0.06 and 4.4 if fitted to confirmed cases, and 0.09 and 3.7 if fitted to the mortality data. For New York, the estimates on the recovery rate $\gamma$ and $R$ are 0.19 and 2.1 if fitted to confirmed cases, and 0.10 and 4.1 if fitted to the mortality data.

time is the time between an individual getting infected and a secondary infection from this individual. Cooper et al. (2020) have applied the $SIR$ model considering data from various countries, including China, South Korea, India, Australia, Italy, the US and Texas.[32] The estimates on the recovery rate are in the range of $0.015 - 0.048$, implying that it takes $20 - 66$ days to recover, and the estimates on the contact rate are in the range of $0.13 - 0.40$, implying a generation time of $2.5 - 7.7$ days. Atkeson (2021) extends the $SIR$ model by adding both the exposed state $E$ and the hospitalized state $H$. Then, using the information from the CDC website, he assumes an infected individual is infectious for 2.5 days and has an average stay in the hospital of two weeks, and a generation time of 4.85 days. Therefore, the recovery time could be as short as $2 - 5$ days or as long as $1 - 2$ months, and the generation time is around $2.4 - 7.7$ days.

Note that all the parameter values mentioned in those studies related to Covid-19 are in daily frequency, and we convert them into quarterly frequency in our simulations. In our baseline simulation, we assume $\gamma = 9$ in quarterly frequency, which implies an individual once infected is infectious for 10 days, or takes 10 days to recover. Then we examine two scenarios-one with a low contact rate of $\alpha = 11.68$ (a generation time of 7.7 days) and the other with a high contact rate where $\alpha = 37.5$ (a generation time of 2.4 days). The estimates from the Imperial College London study (Ferguson et al. 2020) suggests the disease mortality rate is 0.81%, while the lower bound of estimates from some other studies is 0.44% adjusted for age. Thus, we choose $\phi = 0.7\%$ in the baseline case. For Covid-19, the rate of losing immunity is still not clear. In the baseline, we choose $\psi = 0.25$, which implies the immunity lasts for one year. In doing the comparative statics, we look at how the steady states change when we vary the disease related mortality rate $\phi$ and the rate of losing immunity $\psi$. For the demographic parameters, we choose the birth rate $b = 0.005$, that is an annual birth rate of 2%, and a death rate of $d = 0.0031$, that is a life expectancy of 80 years. Note that in the epidemiology models the birth rate and death rates are interpreted as the entry and exit to the population, including biological birth and death, travel, migration, etc. Thus, the estimates using only biological birth and death are very low estimates as during the pandemic many countries including UK and US did not close their borders.

Figure 2 provides the simulation results of the $SIRS$ epidemiological model, described in Sect. 2. We assume here the lockdown measure is exogenously given. It shows how the $SIRS$ model varies when the lockdown measure $\theta$ changes from 0 to 1. There are two scenarios-the solid line depicts the low contact rate $\alpha = 11.68$ case and the dashed line shows the high contact rate $\alpha = 37.5$ case. The contact rate determines the disease prevalence, or the reproduction number $R_0$, shown in the bottom left panel. A higher contact rate implies a larger reproduction number $R_0$. With no lockdown, the reproduction number $R_0$ in the low contact rate scenario is around 1.2, while the reproduction number $R_0$ in the high contact rate scenario is around 4.1. Both of these reproduction numbers lie in the reasonable range of what has been reported for Covid-19. We can see that with a more stringent lockdown (i.e., as $\theta$ increases),

[32] The estimates on the recovery rate and contact rate are 0.035 and 0.35 for China, 0.035 and 0.4 for South Korea, 0.04 and 0.2 for India, 0.05 and 0.19 for Australia, 0.037 and 0.18 for Italy, 0.015 and 0.178 for the US, and 0.048 and 0.13 for Texas.

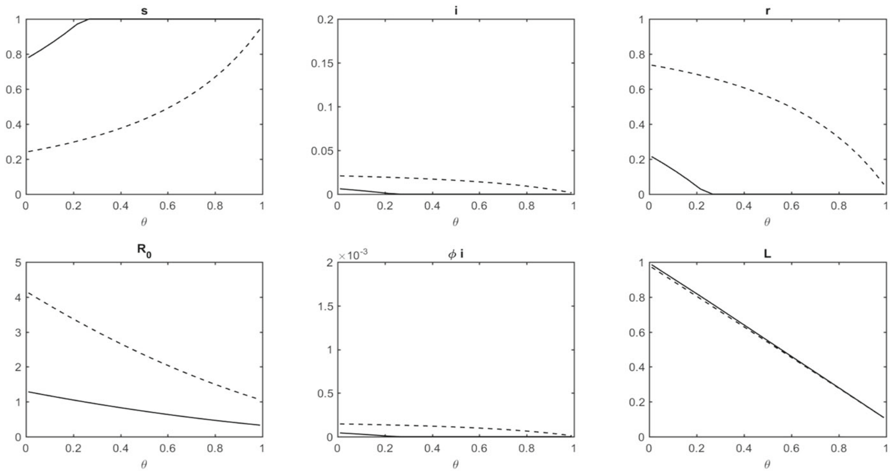

**Fig. 2** The Simulation of the SIRS Epidemiological Models. Note: This figure provides the simulation results of the $SIRS$ epidemiological model described in Sect. 2. There are two scenarios-the solid line is the one with a low contact rate $\alpha = 11.68$ and the dashed line is that with a high contact rate $\alpha = 37.5$. The panels clockwise are the susceptible $s$, the infected $i$, the recovered $r$, the reproduction number $R_0$, the disease related death $\phi i$ and the effective labor force $l$

the reproduction number $R_0$ decreases and the infectious diseases are eradicated when the reproduction number $R_0$ falls below 1.

Furthermore, in Fig. 2 if we compare the fraction of the infected (the top middle panel), the fraction of disease related death (the bottom middle panel) and the effective labor force (the bottom right panel), the difference between the two scenarios is extremely small. When $\theta = 0$, the difference in the fraction of the infected is 1%, the difference in the disease related mortality is 0.01% population, the difference in the effective labor force is 1.5%. However, when we vary the contact rate, there are large differences in terms of the composition of the healthy individuals (susceptible or recovered). We know that higher the contact rate and the reproduction number, the easier it is to transmit the disease and more people move from being susceptible to being infected and then being recovered. Therefore, in the top left panel, the fraction of the susceptible is much larger when the contact rate is smaller, compared with the case when the contact rate is larger. For instance, when there is no lockdown $\theta = 0$, the fraction of the susceptibles is 78% when there is a low contact rate, and 24% when there is a high contact rate. In contrast, in the top right panel, the fraction of the recovered is much smaller when the contact rate is lower compared to the case when the contact rate is higher. For instance, when there is no lockdown $\theta = 0$, the fraction of the recovered is 21% with a low contact rate, and 74% with a high contact rate.

### 5.2 Comparative statics

The following economic parameters are chosen in line with the literature (quarterly frequency): discount rate $\rho = 0.0138$, capital share $\beta = 0.36$, depreciation rate

$\delta = 0.0125$, and the scale parameter in the production function $A$ is normalized to 1. The utility function is of CES form $U(c) = \frac{c^{1-\sigma}}{1-\sigma}$ and we set $\sigma = 1$. In the baseline simulation, we choose the efficacy of lockdown $\zeta = 0.5$ and the productivity of working at home $\xi = 0.1$. Dingell and Neiman (2020) find that in developed countries about 37% of jobs can be done from home while in less developed countries only 10% can be done from home. Productivity of these jobs is likely to be lower than if they were done in the normal workplace (see below) and will vary for different groups.

Figure 3 depicts the function $G$, the net marginal benefit of lockdown measure, varying the lockdown measure $\theta$. The left panel is for the contact rate $\alpha = 11.68$, which implies $\bar{\theta} = 0.23$. Thus, the $G$ function is only shown for $\theta \in [0, 0.23]$. The dashed line is the $G$ function when there is no disutility from disease related mortality ($\chi = 0$). It is negative throughout implying that the optimal policy is to have no lockdown in steady state.[33] The solid line is for the case with the disutility weight $\chi = 15000$. With a larger weight on the disutility of disease related death, the marginal benefit of disease controlling is larger, and it is more likely for there to be a lockdown.

The weight for welfare loss due to mortality, $\chi$, affects the optimal lockdown as it directly affects its marginal benefit through saving lives. What is the welfare trade-off between utility and loss through mortality is a complex issue. In the literature, it is often calibrated using estimates of the value of a year of life, and estimates of the average life remaining of victims.[34]There is a wide range of estimated statistical value of life, from 6 times of annual GDP per capita to 150 times of annual GDP per capita. With their preferred chosen parameter value, Hall et al. (2020) find that a representative agent would be willing to sacrifice 70% of a year's consumption when there is a high death rate and 38% of consumption with a low death rate to avoid deaths from the pandemic. Alvarez et al. (2020) find that the total welfare cost of the pandemic is equivalent to a loss of 30% to 40% of one year GDP. The simulation results of our benchmark case with $\chi = 15000$ are in line with these estimates and if anything on the conservative side. In the steady state, the social planner is willing to sacrifice around 19.8% consumption to control the prevalence of infectious disease, and the total welfare cost of the virus is equivalent to a loss of 27.8% of one year GDP.

The right panel in Fig. 3, is for the case of the contact rate $\alpha = 37.5$, which implies $\bar{\theta} = 1$. Thus, the $G$ function is defined for $\theta \in [0, 1]$. The dashed line is the scenario without any disutility from disease related death ($\chi = 0$), and the solid line is for the case when the disutility weight $\chi = 15000$. Similar to the low contact rate scenario, with a larger disutility weight the marginal benefit of lockdowns is larger. The optimal policy is not to impose any lockdown if there is no welfare loss from disease induced mortality regardless of the severity of the disease prevalence. However, compared with the left panel, with a disutility weight of $\chi = 15000$, the net marginal benefit is still negative. It suggests that in the high contact rate scenario, it is less likely for lockdown, to be imposed compared with the low contact rate scenario. Or in order to impose a lockdown, there needs to be a much larger weight on the welfare loss

33 See the news report from UK by Walters (2021).

34 Eichenbaum et al. (2020) have an estimate close to EPA numbers while Hall et al. (2020) and Holden et al. (2020) use a value of statistical life measure.

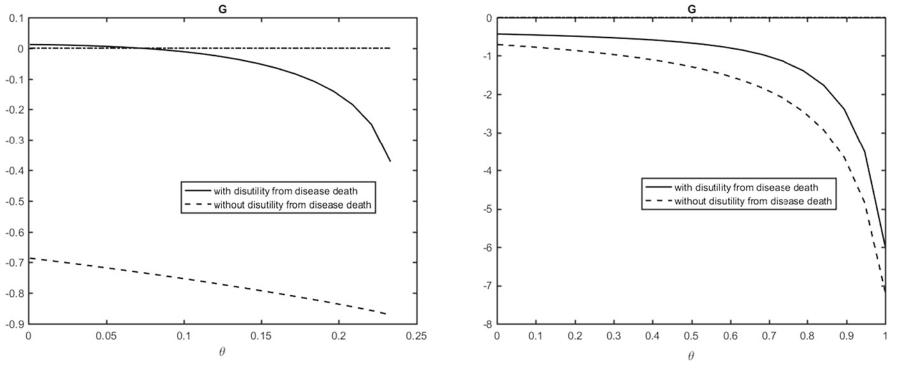

**(a)** $\alpha = 11.68$ **(b)** $\alpha = 37.5$

**Fig. 3** G function-The net marginal benefit of lockdowns, $\theta$. Note: This figure depicts the function $G$, the net marginal benefit of lockdown, varying the lockdown measure $\theta$. The left panel is for the contact rate $\alpha = 11.68$, and the right panel is for the contact rate $\alpha = 37.5$. In each panel, the dashed line depicts $G$ there is no disutility from disease related death ($\chi = 0$), and the solid line is when disease mortality reduces the welfare measure (with $\chi = 15000$)

from the disease related death. At the first sight, this result may seem to be very counter-intuitive. Note that here we only look at what happens in the steady state rather than on the short-run dynamics. If we recall the differences of the two scenarios in the $SIRS$ model, the two economies in the steady state have the similar effective labor supply and the fraction of disease related death. The two economies differ in their composition of the healthy individuals. The economy with a low contact rate has a much large fraction of susceptibles, and thus the marginal benefit of imposing lockdowns is higher. In contrast, in the economy with a high contact rate and thus a larger fraction of the recovered, the marginal benefit of imposing lockdown is smaller as the fraction of susceptibles is smaller. In the context of Covid-19, when a country has a small fraction of its citizen being susceptible to the disease, as the rest of population have gained immunity either by being infected or vaccinations, it is less likely for that country to impose lockdowns.

The state $R$ distinguishes this model from the $SIS$ model. In the $SIS$ model as $\alpha$ increases then lockdowns increase in steady state as the fraction of infected in the population increases without intervention leading to a higher flow of infections and mortality (see Goenka et al. (2020, Figure 5)). However, in the $SIRS$ model, the results are different as indicated above. With an increase in $\alpha$ eventually enough people would have been infected and recovered so that in steady state the fraction of susceptibles is low enough even if infections and mortality are enduring that quarantines are not optimal. This result is for the long run-in the transition to the long run, loss from mortality and infections will be much higher which will have different implications for the optimal quarantines.

For the following comparative statics exercises, we assume that disease related mortality reduces welfare with the weight $\chi = 15000$. As the qualitative results are similar for both scenarios of high and low contact rates, we present the results for the intermediate case of $\alpha = 20$. The baseline parameters imply in the steady state, 55%

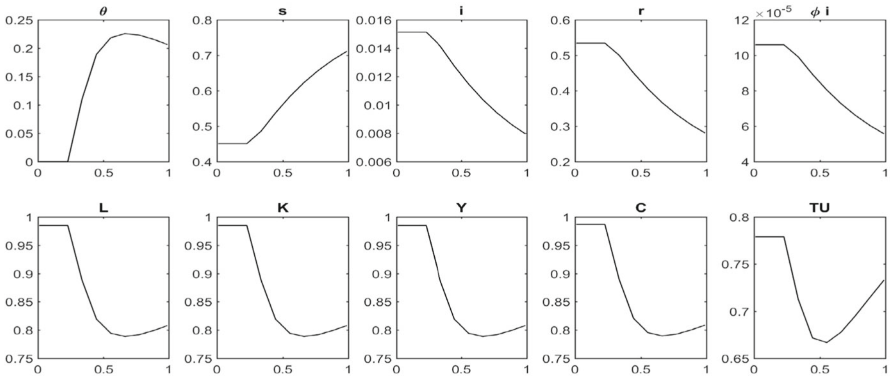

**Fig. 4** The simulation results varying the efficacy of lockdown, $\zeta$. Note: This figure shows the equilibrium steady values of the endogenous variables as compliance or efficacy of lockdown, $\zeta$, is varied of population is susceptible, 1.1% of population is infected and infectious, 43.9% of population is recovered, and 0.008% of population dies as the result of the disease. The effective labor force is 80% of the total population. The steady state capital, output and consumption are also around 80%, compared with the economy in the disease-free steady state. Due to the welfare loss from disease related death, the total welfare is around 67% of the total welfare in the disease-free steady state. For easier comparison, in Figs. 4, 5, 6, 7, all economic variables (labor force, capital, output, consumption and total welfare) are plotted in terms of the fraction of the counterparts in the disease free steady state.

#### 5.2.1 Effects of increasing efficacy of lockdown, $\zeta$

We examine the impact of increasing effectiveness or compliance with quarantine, $\zeta$, on equilibrium steady state values of the endogenous variables in an endemic steady state. Figure 4 shows changes in the steady state, when we vary the efficacy of lockdown from 0 to 1. Initially, as compliance increases lockdowns increase but eventually there is a trade-off between the two. As we would expect, with higher compliance, the optimal policy will be to reduce the extent of lockdown. However, since compliance increases, the infectious disease is better controlled and the fraction of the infected decreases and disease related deaths drop. With a less stringent lockdown and fewer infected individuals, the effective labor force rises, as well as all the other economic variables and total welfare.

#### 5.2.2 Effects of raising productivity of working at home, $\xi$

Now we examine the impact of increasing productivity with quarantine, $\xi$, on equilibrium steady state values of the endogenous variables in an endemic steady state. Figure 5 shows the changes in steady state, when we vary the productivity of working at home, $\xi$, from 0.1 to 0.5. As productivity from working from home increases, the optimal policy is to have more strict quarantines. With stricter quarantines infections

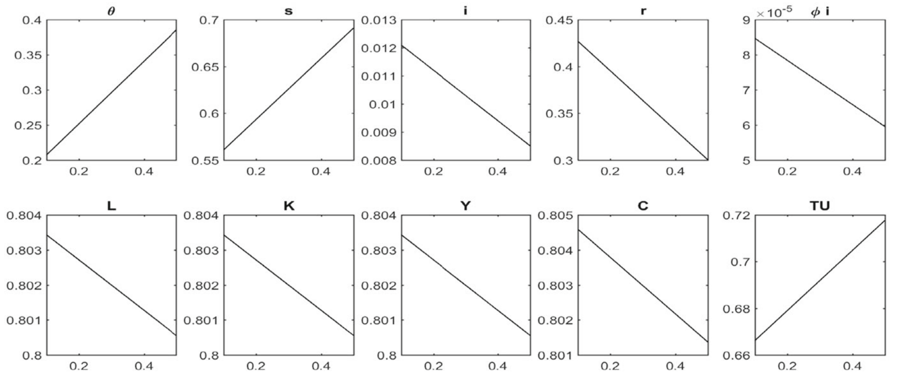

**Fig. 5** The simulation results varying the productivity from working from home, $\xi$. Note: This figure shows the equilibrium steady values of the endogenous variables as productivity from working from home, $\xi$, is varied fall and the economic variables increases and mortality decreases. While this paper has homogeneous households its implications are consistent with the emerging literature on working from home for different segments of the population.[35] The model would suggest that for households who have higher productivity from working at home during a quarantine the infection rates will be lower and the economic outcomes will be better than for those who cannot. This suggests that differentials may emerge across different segments of the population in terms of economic and health impact and they may also have different views on desirability of a quarantines. Soares et al. (2021) show that working from home has a lower distribution of wages in LMICs than developed countries. Our model will predict that we should expect lower quarantines in these countries. This is consistent with the evidence that the poorest countries had the least severe lockdowns (see Gottlieb et al. 2020).

Note that we are plotting the optimal quarantine and outcomes, so for households with low home productivity, while the utility rates are lower and infection rates are higher, the optimal response is still to have lower (or no) quarantines.

#### 5.2.3 Effects of varying disease related mortality rate, $\phi$

In Fig. 6 we show the effect of the disease related mortality rate $\phi$ rising from 0.6 to 1. The increased disease related mortality leads to more stringent lockdowns, and thus a smaller fraction of the infected. Nevertheless, with a high disease mortality rate, the fraction of disease related death rises. The effective labor force decreases, and thus capital, output and consumption all decrease. With both a lower consumption level and higher mortality, the total welfare falls. Treating consumption as endogenous helps understand the effect of having a fully general equilibrium model where capital is modelled. If we look at the panels in the second row of Fig. 6 we see that capital, labor, output and consumption decrease relative to the disease free steady state. Thus,

[35] See Brown and Ravallion (2020), Lekfuangfu et al. (2020), Lewandowski et al. (2020); Mongey et al. (2021).

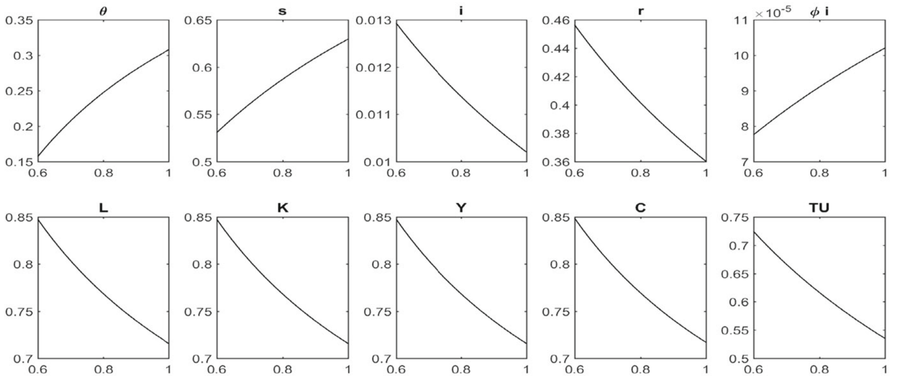

**Fig. 6** The simulation results varying the disease related mortality rate, $\phi$. Note: This figure equilibrium steady values of the endogenous variables as the disease related mortality rate, $\phi$, is varied treating the flow utility as constant as well as not modelling capital will underestimate the effect of the disease as it would only capture the changes in the fractions of population in the different health states in the first row but not changes in output and flow utility.

#### 5.2.4 Effects of varying the rate of losing immunity, $\psi$

Figure 7 shows the changes in steady state, when we vary the rate of losing immunity from $\psi = 0.12$ (2 years of immunity) to $\psi = 0.5$ (6 months of immunity). When the rate of losing immunity is small or the duration of the immunity is long, it is optimal not to have any lockdown, as the fraction of the recovered is large and the fraction of the susceptible group is small. In contrast, when the rate of losing immunity increases, or the duration of the immunity decreases, we need to impose lockdowns, as there are proportion of susceptible individuals rises among the healthy population.The changes in the fraction of both the infected and the disease related death are small, though hump-shaped. Moreover, with larger lockdowns, all economic variables drop. The drop in the total welfare is largely driven by the decrease in consumption. As $\psi$ is varied the model captures the escape from immunity. Thus, as $\psi = 0$ the model reduces to the $SIR$ model. In this case, in the long run lockdowns will not be necessary as the fraction of recovered is high and the fraction of susceptibles is low. It is for this reason that the mutations Delta and Omicron have been especially worrying for long-run control of Covid and many countries imposed pre-emptive measures so that these variants do not take seed in their populations. Figure 7 shows the non-monotonic behavior of $i$ and mortality $\phi i$ in the long run as $\psi$ increases.

#### 5.2.5 Effects of varying the welfare loss weight, $\chi$

In order to explore further the role of the weight on welfare loss from disease related mortality, Fig. 8 shows the changes in steady state, when the weight is varied from $\chi = 0$ to 30000. When there is no weight (or low weight) on welfare loss from disease

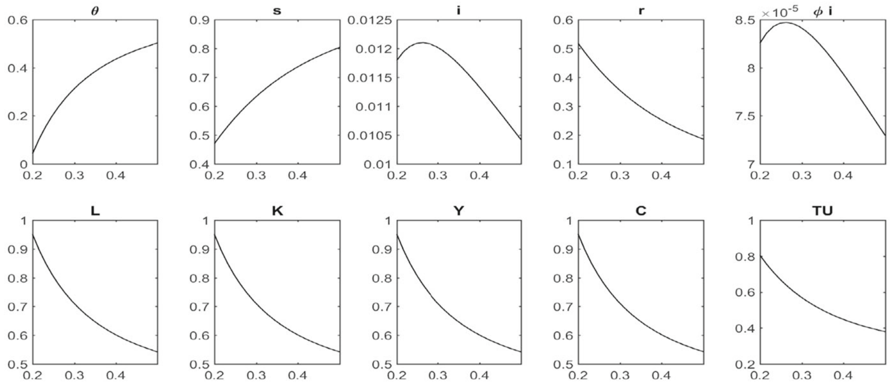

**Fig. 7** The simulation results varying the rate of losing immunity, $\psi$. Note: This figure equilibrium steady values of the endogenous variables as the rate of losing immunity, $\psi$, is varied

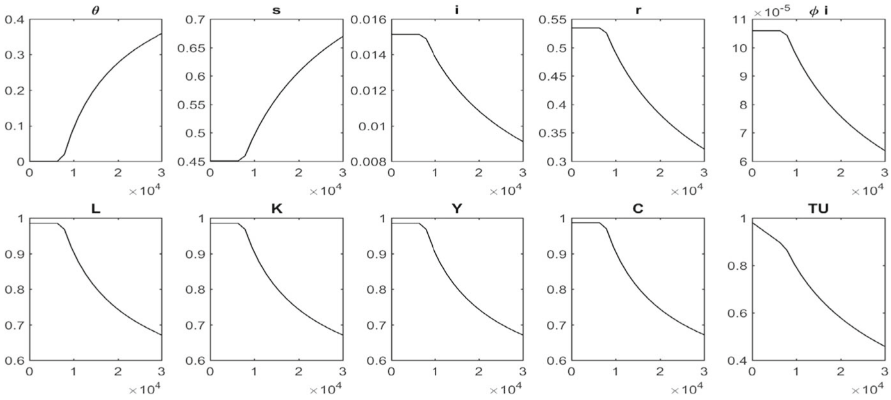

**Fig. 8** The simulation results varying the disutility weight ,$\chi$. Note: This figure shows equilibrium steady values of the endogenous variables as the disutility weight, $\chi$, is varied related death, the optimal policy is no lockdown. When the weight increases, as we would expect, there will be more stricter lockdowns and a better control in disease prevalence (lower infection level and disease related death). However, the economy will have lower levels of output and consumption. The total welfare drops due to both lower level of consumption and larger weight on the disutility of death, despite the fewer number of deaths.

## 6 Transversality and sufficient conditions

In this section we study the sufficiency of the first order conditions with disease related mortality. It is well known in the literature that with $SIS$ or $SIR$ dynamics the constraints are not convex and it is unclear if either the Arrow or the Mangasarian sufficiency conditions will be satisfied (Gersovitz and Hammer (2003)). Goenka et al.

(2014) provided a sufficiency result in a neo-classical framework with SIS dynamics but no disease mortality.[36] Goenka et al. (2020) have sufficiency results for a neo-classical model with $SIS$ dynamics endogenous mortality as this one, Goenka et al. (2021) have it for a model $SIR$ model with endogenous mortality and health capital. While the structure of arguments are similar, they are different as the state variables which generate the non-concavity of the Hamiltonian differ in each of these papers.

We directly show the inequality of local optimality of the Hamiltonian along *any* interior path that satisfies the first order necessary and transversality conditions. This is done by adapting the method of Leitmann and Stalford (1971). As a corollary, the disease endemic steady state will be locally optimal. Optimality of the disease free steady state is not in question as it is the neoclassical steady state.

Denote the state variables $\mathbf{x}_t = (k_t, i_t, s_t, \Lambda_t)$, control variables $\mathbf{z}_t = (c_t, \theta_t)$ and co-state variables $\lambda_t = (\lambda_{1,t}, \lambda_{2,t}, \lambda_{3,t}, \lambda_{4,t})$. Then the Hamiltonian for solving for the equilibrium (9) becomes

$$H(\mathbf{x}_t, \mathbf{z}_t, \lambda_t) = e^{-\Lambda}[u(c) - \chi v(\phi i)] + < \lambda_t, \dot{\mathbf{x}}_t >$$

where $< \mathbf{x}, \mathbf{y} >= \sum_1^n x_j y_j$ is the dot product of two vectors $\mathbf{x} = (x_1, .., x_n)$, $\mathbf{y} = (y_1, ..., y_n)$.

Let $(\mathbf{x}_t^*, \mathbf{z}_t^*, \lambda_t)$ denote the solutions which satisfy the first order necessary conditions (10)-(15) where $\mathbf{x}_0^* = (k_0^*, i_0^*, s_0^*, \Lambda_0^*)$.

The standard transversality conditions are

$$\lim_{t\to\infty} \lambda_{j,t} x_{j,t}^* = 0, \; j = 1, .., 4. \tag{28}$$

This condition holds only at the solution $x_{j,t}^*$ , not for any admissible path $x_{j,t}$. Moreover, $\lambda_t$ is only identified by the FOCs at $(\mathbf{x}_t^*, \mathbf{z}_t^*)$. Many studies in literature on endogenous discounting used a weaker transversality condition due to Michel (1982), originally for a fixed discount rate, where along the optimal paths

$$\lim_{t\to\infty} H(\mathbf{x}_t^*, \mathbf{z}_t^*, \lambda_t) = 0. \tag{29}$$

However, since our model is non-convex with endogenous discounting, this condition is not enough for sufficiency as the framework of the earlier results do not hold. We provide a direct proof of sufficiency by proving the following transversality condition (Cartigny and Michel 2003) for state variables for any admissible $x_t$,[37]

$$\lim_{t\to\infty} \lambda_{j,t}(x_{j,t}^* - x_{j,t}) \le 0. \tag{30}$$

In Appendix A.2, based on the standard transversality conditions (28) and special structure of the model on the convexity in control variables (but not in state variables),

[36] This paper also included the additional state variable health capital which can reduce contact rate and increase recovery rate.

[37] See Goenka, Liu and Nguyen (2021) for further discussion.

and the boundedness of state variables we are able to prove the transversality conditions (29) and (30).

We adapt the method developed by Leitmann and Stalford (1971) for a sufficiency condition to our (non-convex) infinite-horizon optimal control problem for the endogenous discounting problem. To do this, we need to make the following weak assumption as in Lietmann and Stalford (1971). Define the augmented Hamiltonian $\bar{H}(\mathbf{x}_t, \mathbf{z}_t, \lambda_t) = H(\mathbf{x}_t, \mathbf{z}_t, \lambda_t) + \langle \dot{\lambda}_t, \mathbf{x}_t \rangle$.

**Assumption 2** Assume that

$$\bar{H}(\mathbf{x}_t^*, \mathbf{z}_t^*, \lambda_t) \geq \bar{H}(\mathbf{x}_t, \mathbf{z}_t, \lambda_t)$$

This is weaker than assuming concavity of the maximized Hamiltonian as in Arrow's sufficiency condition and the Mangasarian condition that the Hamiltonian is jointly concave in state and control variables (See Remark 2 in Appendix A.2 ).

**Proposition 4** *Consider the maximization problem (8) and suppose that an interior continuous path* $(\mathbf{x}_t^*, \mathbf{z}_t^*)$ *and associated costate variables* $\lambda_t$ *exist and satisfy the first order necessary and transversality conditions (10)-(15) and (28). Then under Assumption 2,* $(\mathbf{x}_t^*, \mathbf{z}_t^*)$ *is a locally optimal solution of (8).*

**Corollary 1** *The disease endemic BGP with lockdown is locally optimal.*

As the endemic steady state with positive lockdown satisfies the necessary conditions, we have shown that it is indeed optimal.

Using the special structure of the autonomous problem we show that $\lim_{t\to\infty} \langle \lambda_t, \mathbf{x}_t^* - \mathbf{x}_t \rangle \leq 0$. This condition is needed to check (local) optimality of a path that satisfies the necessary conditions. This is crucial as when we check the maximality of the Hamiltonian we can decompose it into two parts: the first just relies on the separability of control and state variables and the concavity in control variables of the objective function, and thus, using standard results the difference between the candidate solution and any other solution is non-negative; and a term that depends on the co-state and the state variables as given above. Recall, the non-concavity in the problem arises from the law of evolution of state variables and the Hamiltonian is also non-concave. As indicated, we show this term converges to a negative value, and we are able to obtain sufficiency of the first order conditions.

The proof for sufficiency is different from that in a $SIR$ model (see Goenka et al. (2021)). In the $SIR$ model there are also two state variables and we need a condition on stability of the disease free steady state that is not needed here. In the current model we are able to establish the relevant transversality condition whether the fraction of infectives, $i$ converges to a positive fraction or zero, or it does not converge. In the $SIR$ model, in a disease endemic steady state, the parametric conditions rule out $i^* \to 0$.

## 7 Conclusion

This paper studied the effect of disease related mortality in an $SIRS$ model where the only way to control the incidence of the disease is via a lockdown which can be interpreted either as an optimally mandated quarantine or a self-imposed isolation chosen by the household. Modelling capital makes the model fully general equilibrium and one can see how flow utility from consumption will change with changes in the parameters of the model. Counter-intuitively, higher infectivity in a $SIRS$ model requires lower lockdowns in a steady state as the fraction of the population which is susceptible decreases. This differs from an $SIS$ framework where lockdowns will increase with infectivity (See Goenka et al. (2020), Figure 5, p. 18) as there is no group who have waning immunity. The optimal quarantine is non-monotonic in compliance or effectivity of the quarantine as higher compliance can be traded off with lower quarantines. The changing population size due to disease related mortality makes discounting endogenous in a model which is non-convex due to disease dynamics, none of the existing sufficiency conditions apply. Using the special structure of the model we directly demonstrate the sufficiency still holds.

**Open Access** This article is licensed under a Creative Commons Attribution 4.0 International License, which permits use, sharing, adaptation, distribution and reproduction in any medium or format, as long as you give appropriate credit to the original author(s) and the source, provide a link to the Creative Commons licence, and indicate if changes were made. The images or other third party material in this article are included in the article's Creative Commons licence, unless indicated otherwise in a credit line to the material. If material is not included in the article's Creative Commons licence and your intended use is not permitted by statutory regulation or exceeds the permitted use, you will need to obtain permission directly from the copyright holder. To view a copy of this licence, visit http://creativecommons.org/licenses/by/4.0/.

## Appendix

### A.1

Given $\theta^{\infty}$, the disease dynamics are given by:

$$\dot{i} = \alpha(1-\zeta\theta)^2(1-i)i - bi - \gamma i - \phi i + \phi i^2.$$

Let $\dot{i} \equiv \Omega$.

We know that there are two steady states when $\Omega = 0$ given by:

$$i^{\infty} = 0, \text{ and } i^{\infty} = 1 - \frac{b+\gamma}{\alpha(1-\zeta\theta)^2 - \phi}.$$

Differentiating, we have

$$\frac{\partial \Omega}{\partial i} = -2[\alpha(1-\zeta\theta)^2 - \phi]i + \alpha(1-\zeta\theta)^2 - b - \gamma - \phi.$$

In a disease free steady state:

$$\left.\frac{\partial \Omega}{\partial i}\right|_{i^{\infty}=0} = \alpha(1-\zeta\theta)^2 - b - \gamma - \phi.$$

Thus, if $\alpha(1-\zeta\theta)^2 - b - \gamma - \phi < 0$ the disease free steady state is stable and if $\alpha(1-\zeta\theta)^2 - b - \gamma - \phi > 0$ it is unstable.

For the disease endemic steady state,

$$0 < i^{\infty} < 1 \Rightarrow 0 < 1 - \frac{b+\gamma}{\alpha(1-\zeta\theta)^2 - \phi} < 1.$$

Checking its stability:

$$\frac{\partial \Omega}{\partial i}\bigg|_{i^{\infty}>0} = -\alpha(1-\zeta\theta)^2 - b - \gamma - \phi.$$

Thus, if $\alpha(1-\zeta\theta)^2 - b - \gamma - \phi > 0$ then the disease endemic steady state exists and is stable, while the disease free steady state is unstable. When $\alpha(1-\zeta\theta)^2 - b - \gamma - \phi < 0$, the disease endemic steady state does not exist and the disease free steady state is stable.

## A.2

It is standard that $0 \leq k_t \leq \max\{k_0, \hat{k}\}$ where $\hat{k}$ is the maximum sustainable capital stock[38]. Then is $c_t$ is bounded by a constant[39] $c_t \leq A$, and hence

$$u(c) - \chi\nu(\phi i) \leq u(A) < +\infty \tag{A.2.1}$$

The proof proceeds via three Lemmas.

**Lemma 1** *We have*

$$\lim_{t\to\infty} \lambda_{4,t}(\Lambda_t - \Lambda_t^*) = 0.$$

***Proof*** Consider any feasible path $(\mathbf{x}_t, \mathbf{z}_t)$ with the same initial condition $\mathbf{x}_0^*$.

It follows from (15) that

$$\lambda_{4,t} = \lambda_{4,0} + \int_0^t e^{-\Lambda_\tau^*}\left[u(c_\tau^*) - \chi\nu(\phi i_\tau^*)\right] d\tau.$$

The transversality condition (30) implies

$$\lim_{t\to\infty} \left[\lambda_{4,0} + \int_0^t e^{-\Lambda_\tau^*}[u(c_\tau^*) - \chi\nu(\phi i_\tau^*)\right] d\tau]\Lambda_t^* = 0.$$

[38] Definition of maximal capital stock is $\hat{k} \in (0, \infty)$ such that $f(k, l) > k$ for all $k \in (0, \hat{k})$ and $f(k, l) < k$ for all $k > \hat{k}$. It implies $k \leq \max\{k_0, \hat{k}\} := \bar{k}$.

[39] If investement is irreversible, then $c_t \leq f(k_t, l_t) \leq f(\hat{k}, 1) := A$. Otherwise, as in Goenka, Liu and Nguyen (2014), we can assume that there exists $\kappa \geq 0, \kappa \neq \infty$ such that $-\kappa \leq \dot{k}/k$ which implies that it is not possible that the growth rate of physical capital converges to $-\infty$ rapidly.

Since $\lim_{t\to\infty}\Lambda_t^* = +\infty$, the identity above is satisfied only if

$$\lambda_{4,0} = -\int_0^t e^{-\Lambda_\tau^*}[u(c_\tau^*) - \chi\nu(\phi i_\tau^*)]d\tau$$

which in turn implies

$$\begin{aligned}\lambda_{4,t} &= -\int_0^\infty e^{-\Lambda_\tau^*}[u(c_\tau^*) - \chi\nu(\phi i_\tau^*)]d\tau + \int_t^0 -e^{-\Lambda_\tau^*}[u(c_\tau^*) - \chi\nu(\phi i_\tau^*)]d\tau\\ &= -\int_t^\infty e^{-\Lambda_\tau^*}[u(c_\tau^*) - \chi\nu(\phi i_\tau^*)]d\tau.\end{aligned}$$

For any $\Lambda$, since $d\Lambda = (\rho - b + d + \phi i)dt$ we have

$$\int_t^\infty e^{-\Lambda_\tau}d\tau = \int_t^\infty \frac{e^{-\Lambda_\tau}d\Lambda_\tau}{\rho - b + d + \phi i_\tau}.$$

Lets denote $q_\tau = \Lambda_\tau$, if $\tau = t$ then $q_t = \Lambda_t$. If $\tau = \infty$ then $q_\infty = \Lambda_\infty = \infty$. Since $0 \le i \le 1$ we get

$$\begin{aligned}&\frac{1}{\rho - b + d + \phi}\int_{\Lambda_t}^\infty e^{-q}dq \le \int_t^\infty e^{-\Lambda_\tau}d\tau \le \frac{1}{\rho - b + d}\int_{\Lambda_t}^\infty e^{-q}dq\\ \Leftrightarrow\ &\frac{e^{-\Lambda_t}}{\rho - b + d + \phi} \le \int_t^\infty e^{-\Lambda_\tau}d\tau \le \frac{e^{-\Lambda_t}}{\rho - b + d}.\end{aligned} \tag{A.2.2}$$

It follows from (A.2.1), (A.2.2) and using l'Hôpital's rule that

$$\begin{aligned}0 &\le \lim_{t\to\infty}\Lambda_t\int_t^\infty e^{-\Lambda_\tau^*}[u(c_\tau^*) - \chi\nu(\phi i_\tau^*)]d\tau \le (u(A) + \chi\nu(\phi))\lim_{t\to\infty}\Lambda_t\int_t^\infty e^{-\Lambda_\tau^*}d\tau\\ &\le (u(A) + \chi\nu(\phi))\lim_{t\to\infty}\frac{\Lambda_t e^{-\Lambda_t^*}}{\rho - b + d}\\ &= \frac{u(A) + \chi\nu(\phi)}{\rho - b + d}\lim_{t\to\infty}\frac{\Lambda_t}{e^{\Lambda_t^*}} = \frac{u(A) + \chi\nu(\phi)}{\rho - b + d}\lim_{t\to\infty}\frac{\dot{\Lambda}_t}{\dot{\Lambda}_t^* e^{\Lambda_t^*}}\\ &= \frac{u(A) + \chi\nu(\phi)}{\rho - b + d}\lim_{t\to\infty}\frac{\rho - b + d + \phi i}{\rho - b + d + \phi i^*}\frac{1}{e^{\Lambda_t^*}} = 0\end{aligned}$$

because

$$\frac{\rho - b + d}{\rho - b + d + \phi} \le \frac{\rho - b + d + \phi i}{\rho - b + d + \phi i^*} \le \frac{\rho - b + d + \phi}{\rho - b + d} \text{ and } e^{\Lambda_t^*} \to \infty \text{ as } t \to \infty.$$

Therefore, for any feasible $\Lambda_t$,

$$\lim_{t\to\infty}\lambda_{4,t}\Lambda_t = -\lim_{t\to\infty}\Lambda_t\int_t^\infty e^{-\Lambda_\tau}[u(c_\tau) - \chi\nu(\phi i_\tau)]d\tau = 0. \tag{A.2.3}$$

Together with (30) we have

$$\lim_{t\to\infty} \lambda_{4,t}(\Lambda_t - \Lambda_t^*) = 0.$$

Note that , since $\lim_{t\to\infty} \Lambda_t = \infty$ so from ( A.2.3) we get $\lim_{t\to\infty} \lambda_{4,t} = 0$. □

**Lemma 2** *We have*

$$i)\ \lim_{t\to\infty} \lambda_{1,t}(k_t^* - k_t) \le 0,$$
$$ii)\ \lim_{t\to\infty} \lambda_{2,t}(s_t^* - s_t) = 0,$$
$$iii)\ \lim_{t\to\infty} \lambda_{3,t}(i_t^* - i_t) = 0.$$

***Proof*** (i) From (10) we get $\lambda_1 \ge 0$. Therefore $\lambda_{1,t} k_t \ge 0$ and (28) implies

$$\lim_{t\to\infty} \lambda_{1,t}(k_t^* - k_t) \le 0.$$

(ii) If $s^\infty = 0$ then it follows from (1) that $b + \psi(1 - i^\infty) = 0$ which is impossible as $b > 0$. Therefore either $s^*$ converges to a positive steady state or the sequence lies in the unit interval and does not converge to zero. Hence, it follows from (28) that

$$\lim_{t\to\infty} \lambda_{2,t} = 0. \tag{A.2.4}$$

As $s$ is bounded, we have

$$\lim_{t\to\infty} \lambda_{2,t}(s_t^* - s_t) = 0.$$

(iii) Note that if $i^*$ converges to a positive steady state or the sequence lies in the unit interval and does not converge to 0, then similar to (ii) we get the conclusion. We just need consider the case $i^*$ converges to zero. It follows from the FOC (11) with interior solutions that

$$\lambda_1 \frac{f_2(k^*, (1 - \theta^* + \xi\theta^*)(1 - i^*))(1 - \xi)}{2(1 - \zeta\theta^*)\zeta\alpha} = s^* i^* (\lambda_2 - \lambda_3) \to 0 \tag{A.2.5}$$

by the transversality condition (28).

Because $0 < \theta^*, l^* = (1 - \theta^* + \xi\theta^*)(1 - i^*) < 1, 2(1 - \zeta\theta^*)\zeta\alpha < 2\zeta\alpha$ then

$$\frac{f_2(k^*, (1 - \theta^* + \xi\theta^*)(1 - i^*))(1 - \xi)}{2(1 - \zeta\theta^*)\zeta\alpha} > \frac{f_2(k^*, 1)(1 - \xi)}{2\zeta\alpha}.$$

When $l = 1$, then problem becomes a neoclassical model, our standard assumptions on production $f$ implies $k^*$ converges to a positive steady state thus $\frac{f_2(k^*,1)(1-\xi)}{2\delta_1\alpha} > 0$

as $t \to \infty$. Therefore, (A.2.5) implies

$$\lim_{t\to\infty} \lambda_{1,t} = 0. \tag{A.2.6}$$

On the other hand, as $\theta^* \leq 1$ we have

$$\frac{f_2(k^*, (1-\theta^*+\xi\theta^*)(1-i^*))(1-\xi)}{2(1-\zeta\theta^*)\zeta\alpha} \leq \frac{f_2(k^*, l^*)(1-\xi)}{2(1-\zeta)\zeta\alpha}$$

Since $l^* = (1-\theta^*+\xi\theta^*)(1-i^*) \geq \xi(1-i^*)$ we get $i^* \geq \frac{\xi - l^*}{\xi}$.
Therefore

$$\begin{aligned}
0 \leq \lambda_2 - \lambda_3 &= \lambda_1 \frac{f_2(k^*, (1-\theta^*+\xi\theta^*)(1-i^*))(1-\xi)}{2(1-\zeta\theta^*)\zeta\alpha s^* i^*} \\
&\leq \lambda_1 \frac{f_2(k^*, l^*)(1-\xi)}{2(1-\zeta)\zeta\alpha s^* i^*} \leq \lambda_1 \frac{f_2(k^*, l^*)(1-\xi)\xi}{2(1-\zeta)\zeta\alpha s^*(\delta_2 - l^*)} \\
&\leq \lambda_1 \frac{f_2(k^*, l^*)(1-\xi)\xi}{2(1-\zeta)\zeta\alpha s^*(\xi - l^*)}.
\end{aligned} \tag{A.2.7}$$

Suppose that $l^* \to \xi$. Because $i^{\infty} = 0$ (disease free ) we have $\xi = l^{\infty} = 1-\theta^{\infty}+\xi\theta^{\infty}$ which implies $\theta^{\infty} = 1$ (full lockdown) because $\xi < 1$. This scenario does not make sense as at the steady state where there is no disease there is a full lockdown. Hence, $2(1-\zeta)\zeta\alpha s^*(\xi - l^*)$ could not converge to zero. Moreover, as $i^{\infty} = 0$ and $\xi > 0$, $l^* = (1-\theta^*+\xi\theta^*)(1-i^*)$ could not converge to zero. Hence, $\frac{f_2(k^*,l^*)(1-\xi)\xi}{2(1-\zeta)\zeta\alpha s^*(\xi-l^*)}$ could not converge to infinity.

Therefore, it follows from (A.2.7), (A.2.6) and (A.2.4) that

$$\lim_{t\to\infty} \lambda_{3,t} = \lim_{t\to\infty} \lambda_{2,t} = 0. \tag{A.2.8}$$

Because $i$ is bounded, we have $\lim_{t\to\infty} \lambda_{3,t} i_t^* = \lim_{t\to\infty} \lambda_3 i = \lim_{t\to\infty} \lambda_{3,t}(i_t^* - i_t) = 0$. □

Michel's theorem (Michel 1982) assumes a constant discount rate for the condition (29). We now show that it holds also for endogenous discounting based on the usual transversality conditions,

**Lemma 3** *The usual transversality condition (28) implies the (29 ) transversality condition.*

***Proof*** We have

$$\begin{aligned}
\lim_{t\to\infty} H = &\lim_{t\to\infty} e^{-\Lambda^*}[u(c^*) - \chi v(\phi i^*)] + \lim_{t\to\infty} \lambda_1\{f(k^*, (1-\theta^*+\xi\theta^*)(1-i^*)) \\
&-c^* - \delta k^* - (b-d-\phi i^*)k^*\} \\
&+ \lim_{t\to\infty} \lambda_2\{b - bs^* - \alpha(1-\delta_1\theta^*)^2 s^* i^* + \psi(1-s^*-i^*) + \phi s^* i^*\}
\end{aligned}$$

$$+\lim_{t\to\infty}\lambda_3 i^*\{[\phi+\alpha(1-\zeta\theta^*)^2]s^*-b-\gamma-\phi\}+\lim_{t\to\infty}\lambda_4\{\rho-b+d+\phi i^*\}$$

Using the results of Lemma 1 and Lemma 2 ($\lim_{t\to\infty}\lambda_{1,t}=\lim_{t\to\infty}\lambda_{2,t}=\lim_{t\to\infty}\lambda_{3,t}=\lim_{t\to\infty}e^{-\Lambda^*}=0$) with the fact that $u(c^*)$, $v(\phi i^*)$ and $f$ and all variables are bounded, it implies that the transversality condition (29) is satisfied. □

For $\bar{H}$ we define $M(\mathbf{x}_t,\lambda_t)=\max_{\mathbf{z}_t}\bar{H}(\mathbf{x}_t,\mathbf{z}_t,\lambda_t)$ as the augmented maximized Hamiltonian. We need the following Lemma.

**Lemma 4** *We have $\bar{H}(\mathbf{x}_t^*,\mathbf{z}_t^*,\lambda_t)\geq\bar{H}(\mathbf{x}_t^*,\mathbf{z}_t,\lambda_t)$ for all $\mathbf{z}_t$. In other word, given $\mathbf{x}_t^*$ then $\mathbf{z}_t^*=\arg\max\bar{H}(\mathbf{x}_t^*,\mathbf{z}_t,\lambda_t)$ and thus $M(\mathbf{x}_t^*,\lambda_t)=\bar{H}(\mathbf{x}_t^*,\mathbf{z}_t^*,\lambda_t)$.*

***Proof*** We have

$$\begin{aligned}
&\bar{H}(\mathbf{x}_t^*,\mathbf{z}_t^*,\lambda_t)-\bar{H}(\mathbf{x}_t^*,\mathbf{z}_t,\lambda_t)\\
&\quad=e^{-\Lambda^*}[u(c_t^*)-u(c_t)]-\lambda_1(c_t^*-c_t)\\
&\qquad+\lambda_1[f(k^*,l^*)-f(k^*,\hat{l})]+(\lambda_3-\lambda_2)[(1-\zeta\theta^*)^2-(1-\zeta\theta)^2]\alpha s^* i^*\\
&\quad=\lambda_1[f(k^*,l^*)-f(k^*,\hat{l})]+(\lambda_3-\lambda_2)[D(\theta^*)-D(\theta)]\alpha(1-i^*)i^*
\end{aligned}$$

where $l^*=(1-\theta^*+\xi\theta^*)(1-i^*)$, $\hat{l}=(1-\theta+\xi\theta)(1-i^*)$ and $D(\theta)=(1-\zeta\theta)^2$.

Since $D(\theta)$ is convex, we have

$$D(\theta^*)-D(\theta)\leq D'(\theta^*)(\theta^*-\theta).$$

It follows from (11) that $\lambda_3-\lambda_2\leq 0$ as we consider interior solutions. Therefore

$$\begin{aligned}
(\lambda_3-\lambda_2)[D(\theta^*)-D(\theta)]&\geq(\lambda_3-\lambda_2)D'(\theta^*)(\theta^*-\theta)\\
&=-(\lambda_3-\lambda_2)2(1-\zeta\theta^*)\zeta(\theta^*-\theta).
\end{aligned}\tag{A.2.9}$$

On the other hand, since $f(k,l)$ is concave with respect to $k$ and $l$,

$$f(k^*,l^*)-f(k^*,\hat{l})\geq f_2(k^*,l^*)(l^*-\hat{l})=-f_2(k^*,l^*)(1-\xi)(1-i^*)(\theta^*-\theta).$$

Since $\lambda_1\geq 0$,

$$\lambda_1[f(k^*,l^*)-f(k^*,\hat{l})]\geq-\lambda_1 f_2(k^*,l^*)(1-\xi)(1-i^*)(\theta^*-\theta).\tag{A.2.10}$$

As $u(c)$ is concave we have

$$e^{-\Lambda^*}[u(c_t^*)-u(c_t)]\geq e^{-\Lambda^*}u'(c_t^*)(c_t^*-c_t).\tag{A.2.11}$$

It follows from (10),(11), (A.2.9), (A.2.10), and (A.2.11) that

$$H(\mathbf{x}_t^*,\mathbf{z}_t^*,\lambda_t)-H(\mathbf{x}_t^*,\mathbf{z}_t,\lambda_t)$$

$$\geq [e^{-\Lambda^*}u'(c_t^*) - \lambda_{1,t}](c_t^* - c_t)$$
$$-[\lambda_1 f_2(k^*, l^*)(1-\xi)(1-i^*) + (\lambda_3 - \lambda_2)2(1-\zeta\theta^*)\zeta\alpha(1-i^*)i^*](\theta^* - \theta)$$
$$= 0.$$

□

***Remark 2*** Assumption 2 is weaker than the assumption on the concavity of maximized Hamiltonian $M(\mathbf{x}_t, \lambda_t)$ in $\mathbf{x}_t$ as in Arrow's sufficiency condition.

Indeed, assuming $M(\mathbf{x}_t, \lambda_t)$ is concave in $\mathbf{x}_t$: Since $M(\mathbf{x}_t, \lambda_t) \geq \bar{H}(\mathbf{x}_t, \mathbf{z}_t, \lambda_t)$ and by Lemma 4 $M(\mathbf{x}_t^*, \lambda_t) = \bar{H}(\mathbf{x}_t^*, \mathbf{z}_t^*, \lambda_t)$ and

$$\begin{aligned}\bar{H}_{x_{j,t}}(\mathbf{x}_t^*, \mathbf{z}_t^*, \lambda_t) &= H_{x_{j,t}}(\mathbf{x}_t^*, \mathbf{z}_t^*, \lambda_t) + \dot{\lambda}_{j,t}\\ &= -\dot{\lambda}_{j,t} + \dot{\lambda}_{j,t} = 0\end{aligned}$$

we get

$$\begin{aligned}\bar{H}(\mathbf{x}_t^*, \mathbf{z}_t^*, \lambda_t) - \bar{H}(\mathbf{x}_t, \mathbf{z}_t, \lambda_t) &\geq M(\mathbf{x}_t^*, \lambda_t) - M(\mathbf{x}_t, \lambda_t)\\ &\geq < M_x(\mathbf{x}_t^*, \lambda_t), \mathbf{x}_t^* - \mathbf{x}_t^* >\\ &= < \bar{H}_x(\mathbf{x}_t^*, \mathbf{z}_t^*, \lambda_t), \mathbf{x}_t^* - \mathbf{x}_t >\\ &= 0\end{aligned}$$

Also, if the Hamiltonian is jointly concave in state and control variables as in the Mangasarian sufficient condition, we easily get Assumption 2 by the properties of a concave function and the FOCs (10)-(15)

$$\begin{aligned}\bar{H}(\mathbf{x}_t^*, \mathbf{z}_t^*, \lambda_t) - \bar{H}(\mathbf{x}_t, \mathbf{z}_t, \lambda_t) \ &\geq < \bar{H}_x(\mathbf{x}_t^*, \mathbf{z}_\mathbf{t}^*, \lambda_t), \mathbf{x}_t^* - \mathbf{x}_t >\\ &+ < \bar{H}_z(\mathbf{x}_t^*, \mathbf{z}_\mathbf{t}^*, \lambda_t), \mathbf{z}_t^* - \mathbf{z}_t >= 0.\end{aligned}$$

However, in our model, the Hamiltonian is not jointly concave if the welfare function is positive, i.e. if $u(c) - \chi\nu(\phi i) > 0$. (see Remark 1). □

We are now ready to prove Proposition 4.

***Proof*** The results of Lemma 1 and Lemma 2 yield

$$\lim_{t\to\infty} \lambda_{1,t}(k_t^* - k_t) + \lim_{t\to\infty} \lambda_{2,t}(i_t^* - i_t) + \lim_{t\to\infty} \lambda_{3,t}(\Lambda_t^* - \Lambda_t) \leq 0. \quad \text{(A.2.12)}$$

Assumption 2 implies

$$H(\mathbf{x}_t^*, \mathbf{z}_t^*, \lambda_t) - H(\mathbf{x}_t, \mathbf{z}_t, \lambda_t) + < \lambda_t, \mathbf{x}_t^* - \mathbf{z}_t^* > \geq 0. \quad \text{(A.2.13)}$$

Taking integral over (A.2.13) we get

$$\int_0^\infty \{H(\mathbf{x}_t^*, \mathbf{z}_t^*, \lambda_t) - H(\mathbf{x}_t, \mathbf{z}_t, \lambda_t)] + \langle\dot{\lambda}_t, \mathbf{x}_t^* - \mathbf{x}_t\rangle\}dt \geq 0$$

It implies

$$\int_0^\infty e^{-\Lambda^*}[u(c^*) - \chi\nu(\phi i^*)]dt - \int_0^\infty e^{-\Lambda}[u(c) - \chi\nu(\phi i)]dt \geq -\lim_{t\to\infty}\langle\lambda_t, \mathbf{x}_t^* - \mathbf{x}_t\rangle.$$

Therefore, it follows from (A.2.12) that

$$\int_0^\infty e^{-\Lambda^*}[u(c^*) - \chi\nu(\phi i^*)]dt - \int_0^\infty e^{-\Lambda}[u(c) - \chi\nu(\phi i)]dt \geq 0$$

and we get the sufficient condition. □
